# Probing Allosteric Kinase Modulators as Next Game-Changers in Fighting Neurodegeneration

**DOI:** 10.1021/acs.jmedchem.5c03783

**Published:** 2026-04-27

**Authors:** Elena Roggiolani, Michela Rosini, Anna Minarini, Filippo Basagni

**Affiliations:** Department of Pharmacy and Biotechnology, 9296 Alma Mater Studiorum—University of Bologna, Via Belmeloro 6, Bologna 40126, Italy

## Abstract

Kinases represent one of the main targets of interest in recent years, completely revolutionizing small molecule anticancer therapy. Particularly, several medicinal chemistry strategies (e.g., covalent and allosteric inhibitors) allowed overcoming of the challenging selectivity issue, thus enabling the massive clinical translation of kinase inhibitors. However, the same success has not been detected yet for tackling neurodegenerative diseases, despite plenty of experimental evidence regarding kinases’ pivotal roles in onset and development of neurodegenerative processes. In this perspective we highlight the therapeutic potential of allosteric kinase modulators in this respect, by showcasing the developmental processes and neuroprotective properties of CNS-directed allosteric kinases modulators developed so far. Moreover, we present additional kinases whose modulation is related to neuroprotection and featured by validated allosteric modulators not yet exploited in this context. This, along with a critical discussion on main drawbacks and future directions, may foster the development of allosteric kinase modulators in neurodegenerative diseases.

## Graphical abstract

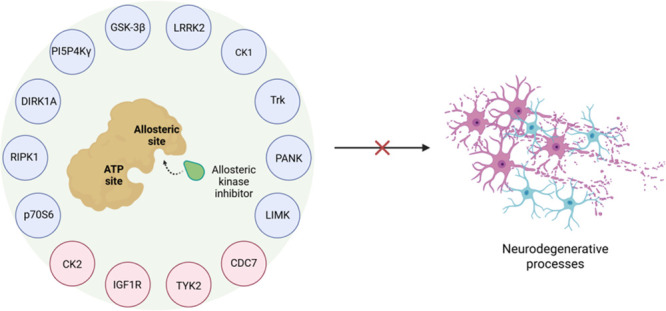

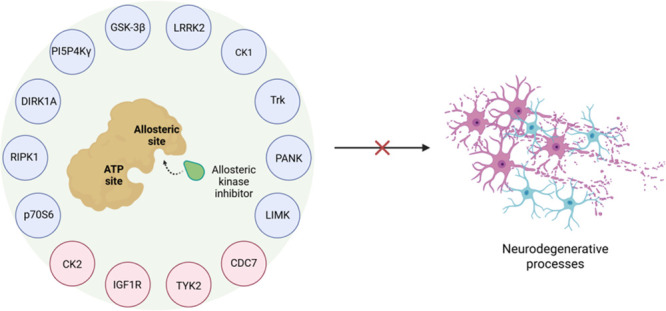

## Significance

- Depicts the overview of allosteric kinase modulators in the context of neurodegenerative diseases, highlighting midterm successes and pitfalls toward new therapeutics
- Outlines alternative kinases that could deserve prospects for future allosteric kinase inhibitors to counteract neurodegeneration
- Provides a forward-looking view by offering a critical perspective on future opportunities that could boost the development of novel kinase therapeutics bearing allosteric mechanism

## Introduction

The search for effective treatments for neurodegenerative diseases has become of utmost importance in recent years due to the continuous increase in life expectancy with the resulting rise in the incidence of such pathologies. In parallel, besides the lack of disease-modifying therapies, the unreliability of early predictive biomarkers paired to the complex and tangled etiopathogenesis strongly demand for developing solid chemical probes which can help in clear target identification.

Kinases represent a ubiquitous protein family that is involved in almost all cellular processes. Although more than 500 protein kinases have been identified in the human genome, around 25% of them remain underexplored and another 23% are considered unexplored, making clear the amount of potential targets that are still not considered.^1^ In neurodegenerative diseases the dysregulation of kinase activity is strictly involved in the onset and development of several neurotoxic pathways, ranging from neuroinflammation, protein aggregates formation and deposition to synaptic dysfunction,^2^ thus fostering the development of kinase inhibitors for potential therapeutic purposes. However, despite plenty of clinical trials, to date, no kinase inhibitor has been approved for neurodegenerative disorder treatment. Since the approval of imatinib more than 20 years ago, kinase-targeting drug discovery programs have revolutionized small molecule-based therapies, reaching 100 kinase inhibitors approved by FDA in 2025, albeit to date most of these are cancer-related.^3^ These discrepancies make roughly evident challenges and weaknesses of the CNS-related drug discovery campaign. Particularly, besides the ordinary BBB permeability issue, solving the selectivity paradigm for CNS-targeted kinase inhibitors seemed to be crucial. In the oncology field, the lack of specificity did not constitute a limit, since many approved drugs are multikinase inhibitors and turned out to be well tolerated. Considering this, 16 FDA-approved kinase inhibitors were evaluated in clinical trials for neurological disorders and more than 70 FDA-approved kinase inhibitors have been tested in animal models of neurological disorders without reaching any clinical translation.^4^ Considering that most kinase inhibitors act in ATP-competitive manner and the high level of sequence similarity within catalytic domains across the kinome as well as the high levels of ATP in the cell, identifying incisive CNS-directed kinase inhibitor without getting into peripheral side effects constitutes an additional developmental hurdle.^5,6^

In recent years several medicinal chemistry tactics have been effectively exploited to develop novel kinase inhibitors with unique kinome-wide selectivity.^5–7^ In this context, allosteric modulation emerged as an alternative effective strategy for developing innovative therapeutics in the field.^8^ Allostery is an intrinsic functional mechanism in cells, where allosteric ligands are able to induce the conformational change of proteins and regulate their biological activities by binding to a site topographically distinct from the orthosteric one. In kinases, the allosteric ligands interact with an allosteric site outside the conserved ATP-binding pocket without direct interaction with the hinge region of the ATP-binding domain. Obviously, they bear significant advantages over ATP-competitive kinase inhibitors with greater selectivity, decreased off-target toxicity, and circumventing resistance mutations. Among all other advantages, they allow the identification of low-affinity drugs because no highly concentrated endogenous substrate-competition occurs, and this is of particular interest for avoiding peripheral side effects with drugs acting at central level.^9^ Furthermore, differently from competitive inhibitors, allosteric ligands enabled the identification of kinase activators or biased modulators, which are considered as essential tools for understanding kinase regulation and mechanisms of action at the cellular level. On the other hand, pocket validation, allosteric ligand identification and its comprehensive biological characterization remain the main challenges in this respect.^10,11^ Currently, many allosteric kinase inhibitors are already marketed with different therapeutic indications, assessing their therapeutic potential. Usually, allosteric kinase inhibitors are noncompetitive and classified depending on the binding site location: type III or type IV if proximal or distal to the ATP-binding site, respectively. More recently, further modalities are gaining particular interest such as allosteric inhibitors binding to the pseudokinase domain of pseudokinase or the extracellular domain of receptor tyrosine kinases.^12^

Through this review, we want to highlight the identification strategies and biological characterization of disclosed allosteric kinase modulators for neurodegenerative disease purposes (i.e., pharmacological tools and therapeutics) with the aim to emphasize their potential, underline successful strategies, and foster the development within this field. Following a glimpse on successful case studies from non-CNS kinase field which may offer proven directions to the neurodegenerative disease’ context, we will focus on the demonstrated ATP and/or substrate noncompetitive modulators with putative allosteric pocket identification and neuroprotective mechanism of action. Each kinase of interest will be addressed according to the purpose for which the allosteric strategy was leveraged (i.e., pursuing selectivity or a biased effect).

Furthermore, the neuroprotective potential of some kinases bearing characterized allosteric pockets or modulators that are not yet evaluated in this respect will also be pointed out. Finally, a critical analysis of reported achievements will be discussed, along with suggesting some alternative strategies that may change the landscape of CNS-directed bioactive compounds with allosteric kinase activity in the near future.

## Allosteric Lessons from Kinases Beyond Neurodegeneration

Therapeutic plans involving allosteric kinase inhibitors have been efficiently adopted in recent years for peripheral pathologies and may therefore pave the way for allosteric strategies in CNS disorders. The turning point was represented by the FDA approval of trametinib as an MEK inhibitor (type III) in 2013, either alone or in combination with dabrafenib (type I), for melanoma treatment carrying BRAF V600E or V600K mutations. To date, the population of approved allosteric MEK inhibitors has increased with cobimetinib in 2015, binimetinib in 2018 and selumetinib in 2020 as therapeutics for unresectable or metastatic melanoma, nonsmall cell lung cancer and neurofibromatosis.^13^ Among several allosteric kinase successful stories, herein we will briefly report on three case studies besides neurodegeneration that prove the potential of this approach in overcoming kinase critical issues.

In 2021 the BCR-ABL1 allosteric inhibitor asciminib (**1**, Figure 1) was approved for chronic myelogenous leukemia (CML) treatment. Previously, other type I or type II BCR-ABL1 inhibitors (e.g., imatinib, dasatinib, nilotinib, and bosutinib) had revolutionized the life of CML patients by increasing the overall survival rate, albeit loss of response or tolerability issues arose in some patients due to drug resistance and off-target effects. From a fragment-based screening on the known allosteric myristate pocket following by iterative hit-to-lead optimization procedures stemmed asciminib (type IV inhibitor), bearing remarkable selectivity and strong antiproliferative activity even against ATP-site mutations models which commonly make ineffective other BCR-ABL1 inhibitors. Furthermore, given that resistance due to myristate pocket mutations is sensitive to ATP-competitive inhibitors, the simultaneous administration of asciminib with type I inhibitors (e.g., nilotinib) dramatically suppress overall resistance, providing a significant advancement in CML therapies.^14^

**Figure 1:**
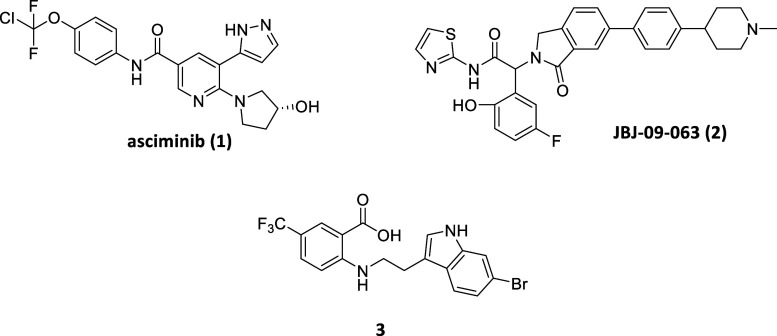
Examples of allosteric kinase inhibitors developed for non-neurodegenerative pathologies.

The approval of different generations of epidermal growth factor receptor (EGFR) tyrosine kinase inhibitors as nonsmall cell lung cancer (NSCLC) therapies was always overshadowed by the arisen resistances: T790 M mutation for gefitinib, erlotinib, and afatinib and C797S mutation for mutant-selective irreversible osimertinib. To overcome these issues a crossed biochemical screening using both wild-type and EGFR^L858R/T790M^ kinases identified a new class of compounds with exquisite potency and selectivity, both kinome-wide and mutant isoforms, able to bind in a new allosteric pocket partially created by the external displacement of αC-helix in kinase inactive conformation.^15^ The main drawbacks of these allosteric EGFR inhibitors turned out to be the loss of efficacy due to the tendency of EGFR mutants to undergo asymmetrical dimerization, thus impeding allosteric binding, and other identified mutations (i.e., L747S), which were effectively overcome when combined with ATP-site inhibitors.^15,16^ Notably, inhibitors targeting this allosteric pocket (e.g., JBJ-09-063, **2**, Figure 1) exerted outstanding in vivo effects in EGFR mutant drug-resistant cancers both as single agent or coadministered with ATP-site-directed inhibitor, resulting in a remarkable synergistic effect.^16,17^ Based on this allosteric approach different chemical modalities were further employed among the years with the aim to empower the therapeutic potential of allosteric EGFR inhibitors (e.g., bivalent ligand or degrader).^18^

Lastly, the discovery and development of allosteric cyclin-dependent kinase 2 (CDK2) inhibitors relied on the structural divergence between its inactive form and that of other similar kinases such as CDK1, despite the high overall structural similarity.^19^ Indeed, a peculiar allosteric pocket (type III) near the C-terminal was identified, characterized and targeted by allosteric inhibitors able to selectively disrupt the interaction of CDK2 and cyclins.^20^ From HTS targeting this pocket and following optimizations resulted the anthranilic acid derivative **3** as the most selective CDK2 inhibitors to date (Figure 1).^21^ **3** demonstrated a strong negative cooperativity with cyclin, stabilizing CDK2 in an inactive conformation. Differently from an ATP-competitive inhibitor, **3** was almost nontoxic in an ovarian cancer cell line due to the high cyclin expression environment, and it showed promise as male contraceptive agents affecting only meiotic CDK2 function.^21^ Further pharmacokinetic refinements were later reported to optimize the therapeutical potential of this series of compounds.^22^

These three stories confirm the efficacy of allosteric modulation in bypassing mutation-induced resistance and achieving functional and kinome selectivity, the main drawbacks of kinase-targeting drug discovery campaigns. Particularly, by leveraging specific kinase conformations, new allosteric regulatory pockets have been exploited to reach an ameliorated pharmacological profile with respect to the corresponding orthosteric ligands. Based on these premises, CNS-targeting kinase modulators will be hereafter addressed to prove how harnessing the allosteric machinery can influence selectivity or a specific biased effect and result in enhanced neuroprotective properties.

## Kinases with Validated Neuroprotective Properties and the Respective Allosteric Modulators: Allosteric Strategies to Pursue a Biased Effect

Differently from conventional orthosteric ligands, allosteric modulators are more prone to the activation with pathway-specific modalities by interacting with usually less-conserved and surface regulatory binding pocket. Particularly, instead of abolishing the complete activity of a target kinase, allosteric modulators can affect specific interactions of the kinase and then modulate a selected pathway while leaving untargeted pathways unaltered. Given that the tuning of activity among the different kinases can greatly change, strategies to interfere with such complex mechanisms are likewise different. Therefore, allosteric kinase modulators offer the intriguingly opportunity to address these exclusive structural features to achieve functional selectivity through different mechanism of actions such as occupying (auto)­inhibitory binding sites (e.g., p70S6), stabilizing (in)­active conformations (e.g., Trk), preventing formation of (in)­active complexes (e.g., LRRK2). However, due to the complexity, the biased modulation is occasionally characterized without elucidating the underlying regulatory mechanism. In this section, kinases of interest will be discussed where the development of allosteric modulators resulted in a specific neuroprotective biased effect.

### Glycogen Synthase Kinase-3β

Glycogen synthase kinase-3β (GSK-3β) is a Ser/Thr kinase which plays a key role in the regulation of multiple signaling cascades in mammalian cells such as mitochondrial functions and metabolism through the modulation of insulin and Wnt/β-catenin pathways. Its activity is regulated by differential phosphorylation of Tyr216 (active form) and Ser9 (inactive form). GSK-3β is constitutively active in cells, and its dysregulation is correlated with several neurological diseases. Particularly, besides acting as pivotal inflammation regulator, pathological aberrant activation of GSK-3β is related to neurotoxic protein aggregation in terms of tau hyperphosphorylation and resulting neurofibrillary tangles (NFT) deposition, or altered amyloid precursor protein (APP) processing and enhanced Aβ-aggregation, besides increased α-synuclein expression.^23^ Based on these premises, several GSK-3β selective inhibitors have been disclosed among the years for potential neurodegenerative disorders treatment, but only two of them reached clinical trials (i.e., lithium ad tideglusib).^24,25^ Notably, also many promising GSK-3β substrate-competitive inhibitors have been reported among the years, demonstrating remarkable neuroprotective and neuromodulatory properties.^26^ Even if the majority of developed compounds have a competitive mechanism, the undesired effects caused by poor selectivity due to the high structural similarity of the orthosteric domain, associated with the involvement of GSK-3β in multiple signaling processes, required the development of selective and possibly biased inhibitors. Particularly, GSK-3β physiologically control β-catenin cytosol level and its prolonged ATP-competitive inhibition was usually related to β-catenin accumulation, nuclear translocation, and activated transcription of oncogenes. In this context, allosteric investigation emerged as suitable strategy to overcome both kinome and functional selectivity.^27^

In 2011 Palomo et al. first performed an extensive computational investigation on 25 different GSK-3β structures retrieved from PDB to identify all potential druggable sites.^28^ By using different pocket identification algorithms, seven conserved pockets were identified: three are the known sites where ATP (Pocket 1), substrate (Pocket 2), and axin/fratide (Pocket 3) usually bind GSK-3β, while four other additional cavities emerged as potential allosteric sites (Figure 2). Among the latter, only ligands for pocket 5, which is located at the protein’s N-terminal lobe, and pocket 7, in the C-terminal lobe, have been reported to date. Particularly, in the same work compound **4** (VP.07, Figure 3A) was identified as a micromolar GSK-3β inhibitor (IC_50_ = 3.01 ± 0.14 μM) with a non-ATP- and nonsubstrate-competitive mechanism of action. From docking studies, the predicted binding site of inhibitor **4** turned out pocket 7, where the 2-oxo-1,2-dihydroquinoline ring resulted sandwiched between Arg209 and Thr235, the carbohydrazide group established H-bond with Ser236, while the side aliphatic tail fitted in the hydrophobic region surrounded by Leu169, Pro331 and Thr330 (Figure 3B). By means of **4**’s biological evaluation, GSK-3β allosteric inhibition has proven to be an alternative promising neuroprotective strategy. Particularly, besides therapeutic potential in Parkinson’s disease (PD) cellular model,^29^ it showed safety and efficacy in in vivo models of multiple sclerosis (MS),^30^ fragile X syndrome (FXS)^31^ and limb girdle muscular dystrophy R1 calpain 3-related (LGMDR1),^32^ highlighting promising perspectives in the treatment of complex neurological pathologies.

**Figure 2:**
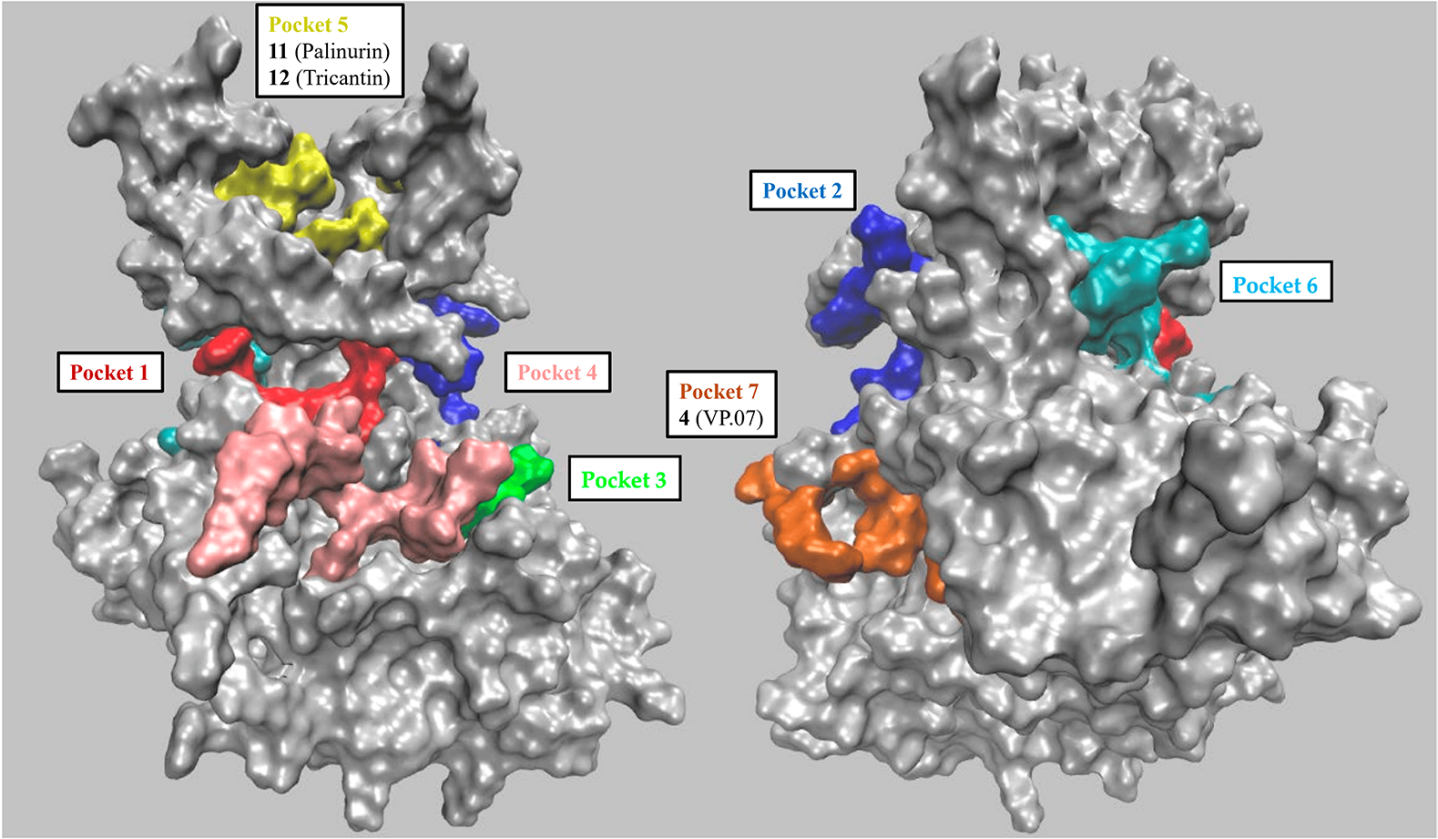
Representation of the main GSK-3β pockets with known highlighted known substrates. The left and right panels contain the front and rear views, respectively. Adapted from Balboni, B.; Masi, M.; Rocchia, W.; Girotto, S.; Cavalli, A. GSK-3β Allosteric Inhibition: A Dead End or a New Pharmacological Frontier? *Int. J. Mol. Sci.* **2023**, 24 (8). DOI: 10.3390/ijms24087541.^27^ Licensed under CC BY 4.0.

**Figure 3:**
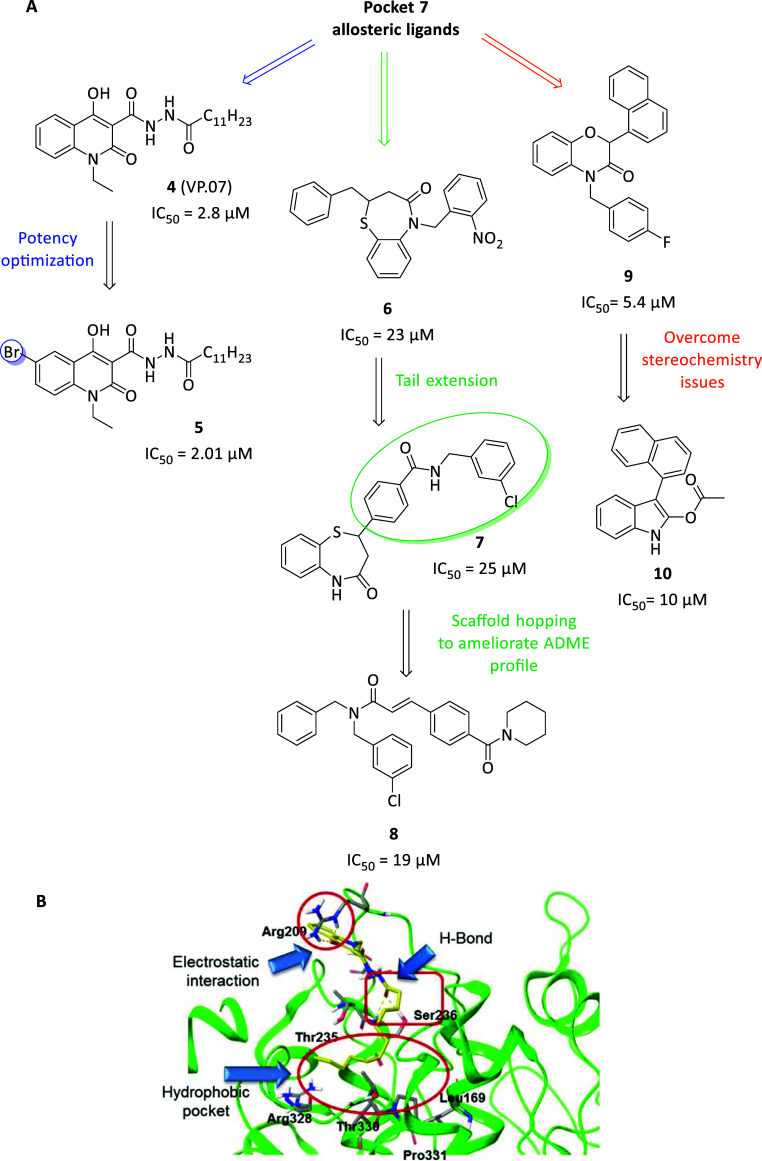
(A) Chemical structures and biological activities of GSK-3β allosteric inhibitors targeting pocket 7. (B) Suggested binding mode for compound **4**. Reprinted in part with permission from Palomo, V.; Perez, D. I.; Roca, C.; Anderson, C.; Rodrìguez-Muela, N.; Perez, C.; Morales-Garcia, J. A.; Reyes, J. A.; Campillo, N. E.; Perez-Castillo, A. M.; et al. Subtly Modulating Glycogen Synthase Kinase 3 β: Allosteric Inhibitor Development and Potential for the Treatment of Chronic Diseases. *J. Med. Chem.* **2017**, 60 (12), 4983–5001. DOI: 10.1021/acs.jmedchem.7b00395.^33^ Copyright 2017 American Chemical Society.

Subsequent structure–activity relationship studies were conducted on compound **4**, reporting low structural tolerance in this pocket for chemical optimization, except for halogen substituents in the aromatic portion.^33^ Namely, 6-bromo insertion in compound **5** ameliorated **4**’s selective potency (IC_50_ = 2.01 ± 0.18 μM, Figure 3A), confirming the noncompetitive mechanism for ATP and substrate of parent compound. To note, upon binding of this class of compounds in pocket 7, mobility restriction occurred in the activation loop, thus altering the substrate binding and plausibly accounting for the allosteric inhibition mechanism. Furthermore, differently from orthosteric competitive inhibitors, they reduced the aberrant activity of GSK-3β without interfering with the β-catenin signaling pathways, whose dysregulation is often referred to prolonged GSK-3β inhibition. Finally, the potential of GSK-3β allosteric inhibition was evaluated in two GSK-3β-associated neuromuscular diseases, such as congenital myotonic dystrophy type 1 (CDM1) and spinal muscular atrophy (SMA). In this case, compound **5** (Figure 3A) improves delayed myogenesis in primary myoblast from skeletal muscle of patients with CDM1 in a dose-dependent manner while both **4** and **5** promoted the survival of SMA motor neurons without registered toxicity after chronic treatment (tested for 7 days).^33^

Albeit not evaluated for neurodegenerative pathologies, simply changing the central core into a benzothiazinone (BTO) with the same ethyl and long side chain maintained micromolar allosteric inhibitory properties. Interestingly, in that case, by introducing a benzyl ring into the thiazinone nitrogen the efficacy completely shifted toward a substrate competitive inhibition mechanism.^34^ Furthermore, a slight stiffening in the side chain increased the activity in the low micromolar range.^35^

Another virtual screening on potential pocket 7 binders led to the identification of benzothiazepinone (BTZ) family as noncompetitive ATP and substrate GSK-3β inhibitors.^36^ Compound **6** (Figure 3A) emerged as the most potent of the series (IC_50_ = 23.0 μM), also displaying good selectivity in a small kinase panel assay. Analogously to the 2-oxo-1,2-dihydroquinoline series, compound **6** located between residues Arg209 and Ser236 and its BTZ ring engaged with Arg209 by pi–cation interaction. In addition, two hydrogen bonds occurred between the carbonyl function and Arg209 as well as nitro group with Ser236. Finally, C2-substituted group of the inhibitor extended in the usual hydrophobic region (Leu169, Pro331 and Thr330). Further BTZs with prolonged side chain in C2 were reported, maintaining a micromolar GSK-3β allosteric inhibition (**7**, IC_50_ = 25 μM, Figure 3A) with promising anti-inflammatory properties.^37^

Within the BTZ series good activity values were achieved, but the predicted ADME profile was not optimal, therefore to improve their drug-like properties the same authors performed a scaffold hopping strategy that lead to the new *N*,*N*-dibenzylcinnamamide (DBCA) series (Figure 3A).^38^ Only compound **8** (Figure 3A) ameliorated the potency reached with the BTZ series, achieving an IC_50_ value of 19 μM with the same mechanism of action. The performed docking analyses revealed the preferential allocation between residues Arg209, Gly210, Thr235 and Ser236, which constitute the same binding pocket 7. In macrophages cell line compounds **8** and **7**, used as reference, reduced pro-inflammatory cytokines IL-1β and IL6 expression upon inflammatory stimuli, while IL-12 and TNF-α values were not affected.

Structurally similarly to the previously reported BTO and BTZ, a new series of benzoxazinones were reported as allosteric GSK-3β inhibitors. In a project devoted to the identification of multifunctional neuroprotective agents (i.e., dual adenosine kinase and GSK-3β inhibitor^39^), compound **9** (Figure 3A) resulted as one of the most potent GSK-3β inhibitors (IC_50_ = 5.4 μM) thanks to an established network of H-bonds between benzoxazinone nucleus and the side chain of Arg209 and Thr235 within pocket 7. To overcome the stereochemistry issues, by means of a ring contraction strategy, some indole derivatives showed the ability to inhibit GSK-3β with a similar behavior (**10**, IC_50_ = 10 μM, Figure 3A). Both compounds **9** and **10** in neuronal cells demonstrated no cytotoxicity paired to the ability of strongly prevent oxidative stress at lower tested concentrations (i.e., 0.1 and 1 μM). Furthermore, the same authors later reported a series of unsymmetrical squaramides active as allosteric GSK-3β inhibitors in the low micromolar range of particular interest for the potential treatment/prevention of retinal degeneration.^40^

Many other computational investigations were conducted to identify allosteric GSK-3β inhibitors locating in cavity 7, leading to potential binders of various structural nature which then revealed almost inactive after in vitro evaluation.^41^

Besides pocket 7, only one compound able to allosterically inhibit GSK-3β through different cavity interactions has been reported. Particularly, in 2005 it was first reported the potent inhibitory activity of the isopropanolic extract from marine sponge Irina variabilis. Following purifications identified the furanosesquiterpenoid palinurin (**11**, IC_50_ = 4.5 μM) and its metabolite, tricantin (**12**, IC_50_ = 7.5 μM), as the active ingredients (Figure 4A).^42^ Both compounds revealed to act as cell-permeable ATP noncompetitive inhibitors with the ability to reduce tau phosphorylation in different cell cultures.^25^ After extensive molecular dynamics simulations, pocket 5, the allosteric site located at the *N*-terminal lobe, was identified as its most suitable binding site through a salt-bridge and a hydrogen bond with the deprotonated hydroxyl of the tetronic ring with Lys86 and a hydrogen bond between Tyr56 and the ester’s carbonyl group (Figure 4B). Furthermore, upon binding, palinurin induced a reduced conformational flexibility of the glycine-rich loop that remains closed in the upper side of the ATP binding cavity, thus reducing the accessibility of the γ-phosphate from the substrate-binding site, plausibly accounting for its allosteric inhibition mechanism.^43^

**Figure 4:**
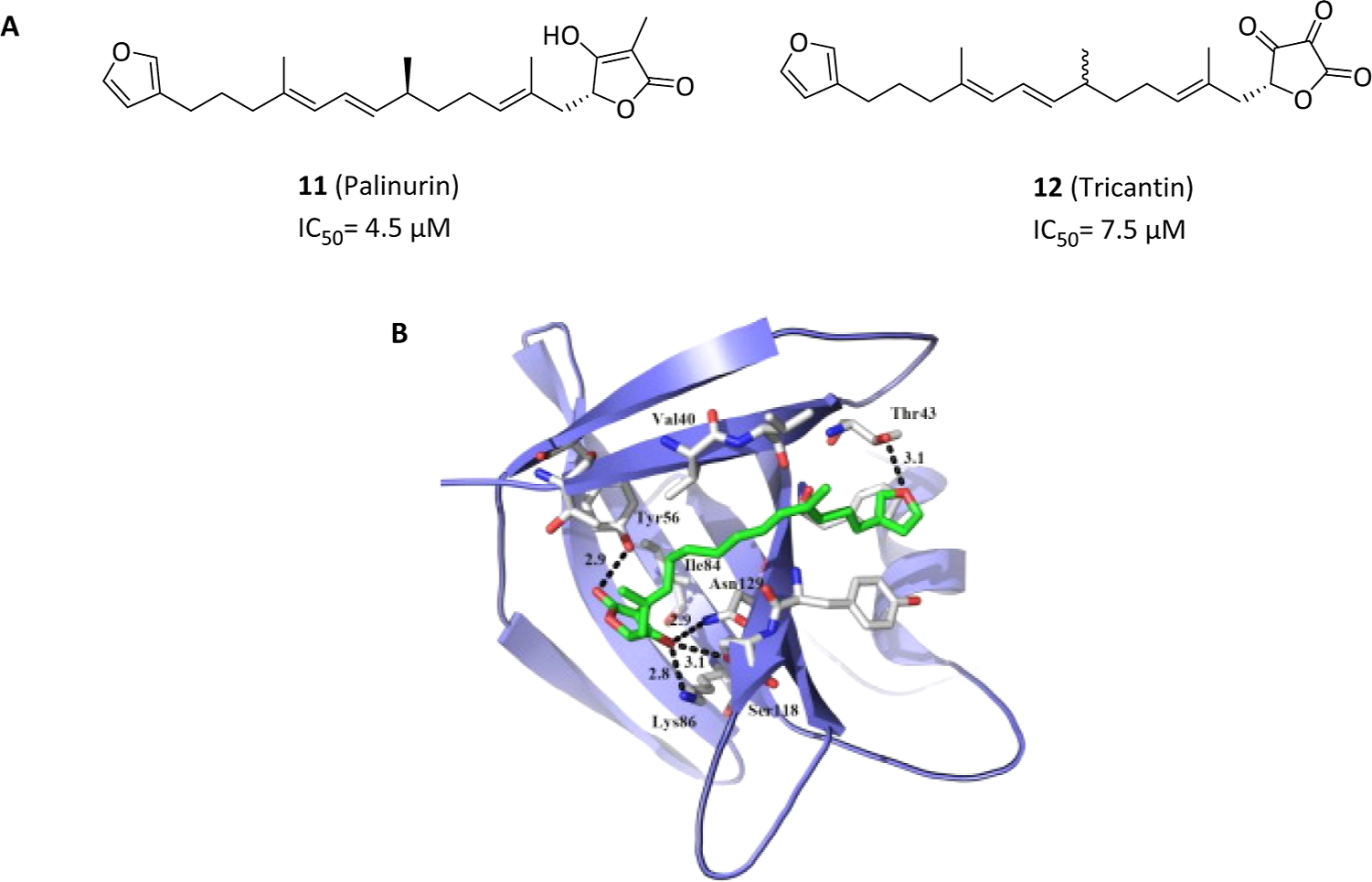
Chemical structures and biological activities of GSK-3β allosteric inhibitors targeting pocket 5 (A) with highlighted docking pose of **11** within the binding site (B). Reprinted in part from Bidon-Chanal, A.; Fuertes, A.; Alonso, D.; Pérez, D. I.; Martínez, A.; Luque, F. J.; Medina, M. Evidence for a new binding mode to GSK-3: allosteric regulation by the marine compound palinurin. *Eur. J. Med. Chem.* 2013, 60, 479–489. DOI: 10.1016/j.ejmech.2012.12.014 with permission from Elsevier.^43^

### Leucin-Rich Repeat Kinase 2

Leucin-rich repeat kinase 2 (LRRK2) is a complex protein composed of four protein–protein interaction (PPI) domains and others two endowed with kinase (i.e., Ser/Thr kinase) and GTPase (mediated by the Ras of complex protein, Roc) activity.^44^ Mutation on LRRK2 gene is one of the main causes in the development of both sporadic and idiopathic PD, resulting in abnormal kinase activity.^45^ Furthermore, LRRK2 exists both as monomers, preferentially localized in the cytosol, and homodimers, preferentially in the membranes, but in the LRRK2 PD variants the equilibrium between the two isoforms is impaired with an abundance of the dimerized form which is related to the neurotoxic kinase hyperactivity.^46^

Several brain-penetrant and ATP-competitive LRRK2 inhibitors have been reported with promising in vivo neuroprotective effects, but almost always related to increase microtubules recruitment, mislocalization, leading to altered vesicular trafficking and cellular dysfunctions.^44^ Therefore, an allosteric inhibition resulted a promising alternative because acting through different mechanisms, such as disruption of dimerization or binding to non-ATP sites, which may not prompt to the same side effects.^47^

5′-Deoxyadenosylcobalamin (**13**), one of the physiological forms of vitamin B12 resulted a LRRK2 inhibitor with a mixed-type mechanism, binding in an allosteric site and affecting the proper ATP binding.^48^ Emerging from a high throughput screening (HTS) upon a FDA-approved compounds library, **13** revealed a micromolar inhibitory activity (IC_50_ = 1.2 μM). Interestingly, also the other B12 forms, which only differ for the (β)-coordinating ligand, maintained the same activity range, underlining low importance for this portion, while in cells, **13** resulted as the only active. From biophysical investigations it should act by modifying the conformational shape of LRRK2 and ATP binding, preferably binding to the dimer and shifting the equilibrium toward the kinase inactive monomeric form. One of the main hallmarks of PD pathogenesis is degeneration of dopaminergic neurons; therefore, **13** was evaluated in three different PD animal models for preserving dopaminergic functions. First, in *C. elegans* recurring specific LRRK2 mutations, **13** restored the loss of dopaminergic functions while ATP-competitive inhibitors are ineffective, supporting the distinct mechanism of **13** in blocking LRRK2 activity and LRRK2-associated neurodegeneration from ATP-competitive inhibitors. Furthermore, it prevented LRRK2-related induced neurotoxicity in the *D. melanogaster* model of PD in terms of rescuing visual response after 2.5 μM **13** treatment. Finally, the administration of **13** in PD mouse model caused a dose-dependent inhibition of LRRK2 autophosphorylation and suppression in the frequency of apoptotic neurons in striatal slice lysates, as well as almost recovered the induced impairment of dopaminergic neurons.^48^

In 2021 another type of potential allosteric inhibitor for LRRK2 kinase was reported, consisting of peptides modified to contain an all-hydrocarbon constrained macrocycle to serve as protein–protein interaction (PPI) disruptors to block the dimer interface. These compounds were designed to specifically target the RocCOR interface domain, which is one of the main involved in mediating the dimerization process. Particularly, after predicting the amino acids involved in PPI within these sequences, peptides were built, maintaining these amino acids and inserting modified olefinic amino acids in positions not crucial for dimerization procedures, with the ultimate goal to form hydrocarbon macrocycles for improving cell permeability. Two different constrained peptides turned out as the best: **14** (LRIP4) targeting the Roc domain and **15** (LCIP1) toward the COR domain. **14** demonstrated to permeate cells, block the dimer formation and the kinase activity both in vitro and in cells, while **15** showed relatively lower cell permeation and binding to the target kinase. Moreover, disruption of the dimerization process resulted in mitigated kinase activity, both in terms of autophosphorylation or Rab phosphorylation, and no induced mislocalization, maintaining the usual cytoplasmic distribution in contrast to filament-like structures occurring with an orthosteric inhibitor. This peculiar mechanism of action resulted in an overall downregulation of LRRK2-mediated ROS production and neuronal apoptosis.^49^

The LRRK2 GTPase activity has been directly related to its pathogenicity, and therefore, some GTP-binding compounds were developed as LRRK2 allosteric inhibitors with potential neuroprotective activity. First, from virtual and biological screening emerged compound **16** (Figure 5) for its ability to reduce LRRK2 GTP binding (i.e., up to 90% at 10 nM) and the resulting kinase activity.^50^ A preliminary evaluation in a neuroblastoma cell line confirmed its ability to suppress mutant LRRK2-induced neuronal degeneration at the nanomolar level. After 20 mg/kg treatment in a mouse neuroinflammation model, besides confirming a significant reduction in LRRK2 GTP-binding and phosphorylation after 1 h i.p. injection, **16** attenuated LPS-induced microglia activation and LRRK2 upregulation. To increase the BBB permeability, compound **17** (FX2149, Figure 5) was properly developed and it maintained all the in vitro activities reported for the parent compound.^51^ In mice brains, **17** was almost bioequivalent of **16** at half dose (i.e., 10 mg/kg vs 20 mg/kg) both in reducing LRRK2 GTP binding, kinase activity inhibition, as well as microglia activation and LRRK2 upregulation in the neuroinflammation model. Further insights in different cellular models with this class of compounds confirmed the peculiar neuroprotective efficacy of GTP-binding inhibitors for PD treatment.^52^

**Figure 5:**
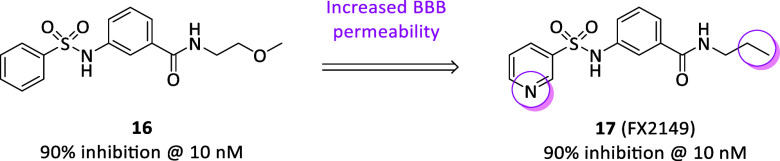
Chemical structures and biological activities of LRRK2 allosteric inhibitors.

### Tropomyosin Receptor Kinase

Tropomyosin receptor kinases (Trk) belong to the family of receptor Tyr kinases and can be distinguished into TrkA, TrkB and TrkC. Their activities are mainly regulated through the binding of neurotrophins (NTs) including NT-3, NT-4/5, the nerve grow factor (NGF) and the brain-derived neurotrophic factor (BDNF). Neurotrophins signaling plays a crucial role in neurogenesis, synaptogenesis and synaptic plasticity,^53^ whereas in neurodegenerative disorders both the decrease in BDNF levels and the impairment in the NGF signaling can be observed.^54^ Moreover, decreased TrkA expression leads to neurodegeneration,^55^ TrkB activation is involved in tau dephosphorylation processes^56^ and cognition enhancement^57^ and TrkC KO animal models have been related to deficiencies in glial cells.^58^ Therefore, activation of NT/BDNF signaling through Trk modulation emerged as powerful neuroprotective strategy.^59^ Unfortunately, TrkA, TrkB, and TrkC kinases have no residue difference in the ATP binding site as happens with other kinases; thus, allosteric modulation offered prospect for lack of target-related side effects of orthosteric agonists. Furthermore, besides activating specific biased signaling, with an allosteric modulation spatial selectivity can be achieved by modulating the receptor signaling only where ligand–receptor interaction occurs, rather than the pure agonist-derived widespread receptor activation.^60^ To date, several Trk modulators or coactivators have been reported, but only two different classes have been clearly defined as Trk positive allosteric modulators and are in clinical trials for AD treatment.^61^

Eisai reported the discovery of **18** (E2511), a ‘biased’ positive allosteric modulator of TrkA that binds to the intracellular juxtamembrane region with a *K*_d_ value of 680 nM. Albeit the chemical structure has not been disclosed yet, several in vivo and preclinical data were provided. Treatment with **18** increased ACh levels and cholinergic function in a dose-dependent manner in neuronal cultures and cerebrospinal fluid (CSF), while after 3 months one-daily administration in tau transgenic mice demonstrated reinnervative effects on cholinergic presynapses with improvement in the number of cholinergic neurons.^62^ These neurotrophic effects were mediated via direct binding to TrkA which enhanced its phosphorylation level in the presence of NGF with a different pattern. To note, hyperalgesia or discrepancies in pain-related genes expression were not found, suggesting a biased mechanism of activation compared to common TrkA activators.^63^ In a Phase I clinical trial (NCT04547361), **18** was well-tolerated, demonstrating no adverse-effects and a disease-modifying potential.^61^

AlzeCure Pharma identified Ponazuril (**19**, Figure 6), already used as a veterinary drug for protozoal parasite-related pathologies, as a candidate for repurposing based on its preliminary promising pharmacological modulation of Trk-signaling. In cells compound **19**, also known as ACD855, could activate TrkA and TrkB up to 45% without ligand and further potentiated receptor activation beyond 100% in the presence of NGF or BDNF with an EC_50_ of 1.9 ± 0.4 μM for TrkA and 3.2 ± 1.2 μM for TrkB. Further optimizations led to compound **20** (ACD856, no chemical structure disclosed) which shown an EC_50_ of 382 ± 28 nM for TrkA and 295 ± 35 nM for TrkB.^64^ Furthermore, **19** and **20** had similar potency for TrkC (0.8 and 0.33 μM, respectively), with lower efficacy on off-targets FGFR1 and IGF1R, thus indicating selectivity toward Trk receptors. By means of affinity labeling and surface plasmon resonance experiments, **20** demonstrated to interact with the intracellular domain of TrkA with a resulting increase in the efficiency of the kinase activity of the Trk receptor.^65^ In rat hippocampal slices **19** at 20 μM increased long-term potentiation as did the exogenous application of BDNF 50 ng/mL.^53^ The local administration of the two compounds in the ventral hippocampus of awake rats resulted in increased ACh level, while other neurotransmitters were not affected. In vivo compounds **19** and **20** reversed the scopolamine- and MK-801-induced cognitive impairment, whose effects resulted blocked with a simultaneous TrkB inhibition and additive to the acetylcholinesterase inhibitor. A deepen in vivo characterization was then conducted on **19** and **20** before moving into clinical trials: **19** exerted nootropic effect and reversed the impairment in memory formation in Wistar rats, while **20** revealed marked procognitive effects in C57BL/6J mice. Further biological investigations confirmed cognitive enhancement properties of **20**, providing in vitro and in vivo evidence of neuroprotective and long-lasting effects that contribute to neurotrophic support and increased neuroplasticity.^66^ Finally, in Phase I clinical trial (NCT05077501) compound **20** was well tolerated with good brain permeability and without showing adverse effects, thus allowing the next Phase II planning.^67^

**Figure 6:**
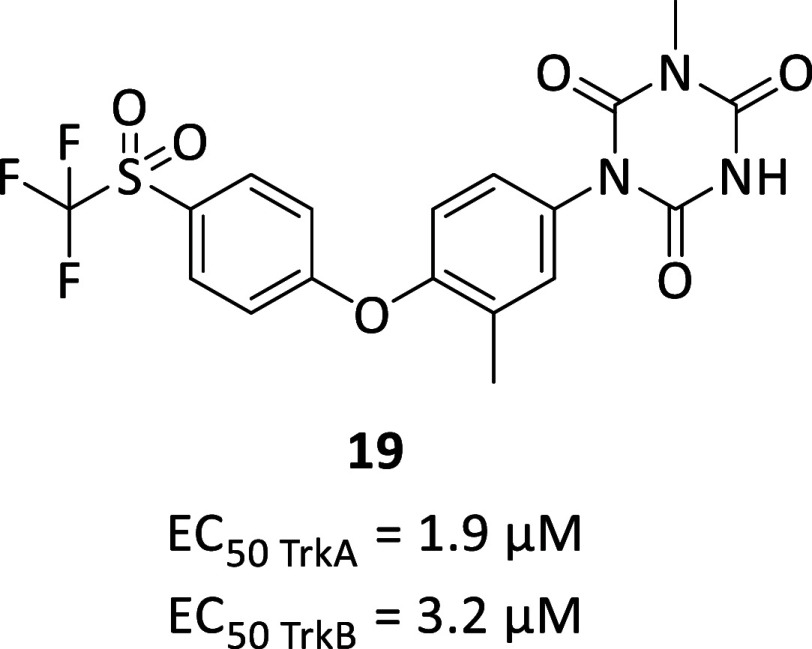
Chemical structure and biological activities of Trk allosteric modulator **19**.

### Pantothenate Kinase

Pantothenate kinase (PANK) phosphorylates pantothenic acid (vitamin B5), thus regulating the first and rate-controlling step in CoA biosynthesis, the major acyl group carrier, and key metabolism regulator. PANK has four different isoforms (i.e., PANK1α, PANK1β, PANK2 and PANK3), encoded by three genes, with different tissue distribution and high structural analogy.^68^ PANK2 represents the primary isoform in neural cells and inactivating mutations in PANK2 gene turns out in pantothenate kinase-associated neurodegeneration (PKAN), an autosomal recessive disorder characterized by progressive parkinsonism, cognitive decline with characteristic iron accumulation in basal ganglia and neuronal CoA deficiency.^69^ At physiological conditions, ATP usually binds PANK in protomer form and activates it in its dimer form, which is able to phosphorylate pantothenate, releasing phosphopantothenate which increases AcCoA concentration. Then, this latter stabilizes PANK in an inactive form through a negative feedback mechanism.^70^

To date, there are not disease-modifying therapies for PKAN with metabolite supplement or iron chelators as the most attempted therapeutic strategies in clinical trials (both discontinued in Phase III).^71^ Alternatively, small molecule PANKs activators represent a promising strategy to compensate for the loss of PANK2 and enhance brain CoA biosynthesis. Exploiting PANK3 as test case, in 2010 it was reported an HTS on a library of compounds with known biological activity to identify putative PANK modulators and it resulted with twenty inhibitors bearing an IC_50_ < 10 μM and eight activators with an EC_50_ < 10 μM.^72^ Emerged inhibitors mainly belong to three different chemical classes: thiazolidinediones and other PPARγ ligands, sulfonylureas, and other insulin secretagogues, or steroids.

Among the identified activators, oleoylethanolamide, oleoyl-carnitine, and tamoxifen demonstrated the higher potency with the ability to reverse AcCoA-mediated PANK3 inhibition. Interestingly, the majority of emerged modulators targeted the AcCoA binding site, but, due to their metabolic instability or promiscuous activity, were discarded and a larger HTS was later reported in 2015.^73^ In this case, the tricyclic compound **21** (Figure 7A) was initially characterized as one of the most potent inhibitors (IC_50_ PANK3 = 25 nM, IC_50_ PANK2 = 92 nM, IC_50_ PANK1β = 70 nM) with a mixed-type inhibitory mechanism verified on PANK3 and, by binding with the ATP-PANK3 complex, able to inhibit CoA biosynthesis in cells.^73^ However, the emerged modulators were not initially assessed as suitable starting hits due to flat SAR and poor solubility. Therefore, a revaluation on the hit list was conducted by filtering with lipophilic ligand efficiency (LipE >2; LipE = pIC_50_ – cLogP), merging both potency and lipophilicity, as primary driving value with the aim to ameliorate the biochemical properties and activity. In this way, starting from the piperazine urea hit compound **22** (PZ-2789, IC_50_ = 844 nM, Figure 7A) and after an extensive SAR campaign (exploiting IC_50_ determination as preliminary ranking index in AcCoA absence), compound **23** was achieved (PZ-2891, IC_50_ = 1.3 nM, Figure 7A) with 800-fold higher potency and better pharmacokinetic properties than parent compound.^74^ In this case, only branched and aliphatic para-substituents in the aniline motif gave efficient PANK modulators, whereas the more flexible acetamido linker was revealed superior to both carbamate and urea ones. Finally, electron-withdrawing substituents in the 3-position of the nicotinonitrile moiety were pivotal for activity and the nitrile group was advanced due to low lipophilicity, while the insertion of an additional nitrogen in the ring close to the nitrile dramatically increased the potency. The so developed series of compounds, called “pantazines”, acted as noncompetitive inhibitors in respect to panthotenate and uncompetitive to ATP through binding to the PANK3•ATP•Mg^2+^ complex across the dimer interface and simultaneously interacting with both PANK3 protomers (Figure 7B).^75^ Particularly, the isopropyl tail locates into a hydrophobic cavity, the carbonyl group interacts with Arg207, while the piperazine ring serves as interprotomer spacer to present the pyridazine to the opposite protomer and engaging Arg306′ with an hydrogen bond and Trp341′ by π–π stacking interactions (Figure 7C). This binding mode of **23** is similar to the pantothenate one, but with the difference that the substrate does not interact with the interface of the dimer. **23** first binds the pantothenate site of PANK3•ATP•Mg^2+^ complex and then closes the flexible loop to obtain the engagement and reorganization of the dimer interface in a locked structure. In this way, at subsaturing concentration, **23** maintained PANK in its active conformation, by preventing the AcCoA binding and its inhibitory feedback mechanism, thus acting as allosteric activator while performed as orthosteric inhibitor in AcCoA absence. At cellular level, the biological outcome is an increased amount of intracellular CoA and a lowered intracellular pantothenate level in a PANK-dependent and selective mechanism. In mice fed with 5 doses of **23** by oral gavage was registered a dose-dependent increase in CoA in liver, forebrain, and hindbrain, even in a PANK2 KO model. Finally, in a mouse model of neuronal CoA deficiency where mice develop normally until day 12.5 then lose weight and had a median life of 52 days, **23**-treated animals gained weight and had a median lifespan of 150 days (with 5/11 mice alive for the entire experiment, 6 months) with a registered increase in CoA in the brain and ameliorated locomotor activity. Due to rapid metabolism limitation, **23** was further overtaken by the cyclopropyl analogue **24** (PZ-3022, IC_50_ = 5.3 nM, Figure 7A), maintaining the same efficacies but still suffering from metabolic liabilities and poor solubility. Finally, **24** set the stage for another round of SAR exploration aiming at optimized PK properties.^76^ In the phenyl ring the cyclopropyl moiety resulted in better activators but less potent binders in comparison to those with the isopropyl moiety, while an inserted fluorine atom in the *ortho*/*meta* position increased the potency and metabolic stability; a methyl group in the piperazine mainly served to increase the solubility, whereas a side chlorine substitution in the pyridazine allowed the best profile. Generally, new derivatives demonstrated excellent oral bioavailability, lower clearance and higher solubility. Furthermore, among the best compounds of the series, **25** (BBP-671, IC_50_ = 1.39 nM, Figure 7A) proved as metabolically stable and BBB permeable with a promising ability to increase CoA levels in mice liver, forebrain, and hindbrain after oral gavage administration. In PKAN mouse model (i.e., carrying neuronal PANK1 and PANK2 gene deletions) **25**, administered as chow supplement maintaining around 10 mg/kg per day, elevated the forebrain CoA concentration and restored hindbrain CoA levels. Furthermore, it led to significant increase in body weight and locomotion, thus providing a solid preclinical foundation for its development as a potential PKAN treatment.^77^ To note, some of these pantazines have been evaluated also for potential treatment of propionic acidemia, an inborn error of metabolism due to propionyl-CoA carboxylase insufficiency which leads to propionyl-CoA accumulation,^78^ and translated into clinical trials (NCT04836494). More recently, development of **25** for PKAN treatment was discontinued due to issues arising in critical dosing determination.^79^ Notably, during the revision process of this manuscript, a new published work focused on newly developed pantazines bearing ameliorated PK and solubility profile (i.e., substituting cyclopropyl moiety with a sulfonamide fragment) revealed that CoA increase at cellular level correlates with difference in affinity between PANK3 and PANK1β, suggesting different isoform’s role.^80^

**Figure 7:**
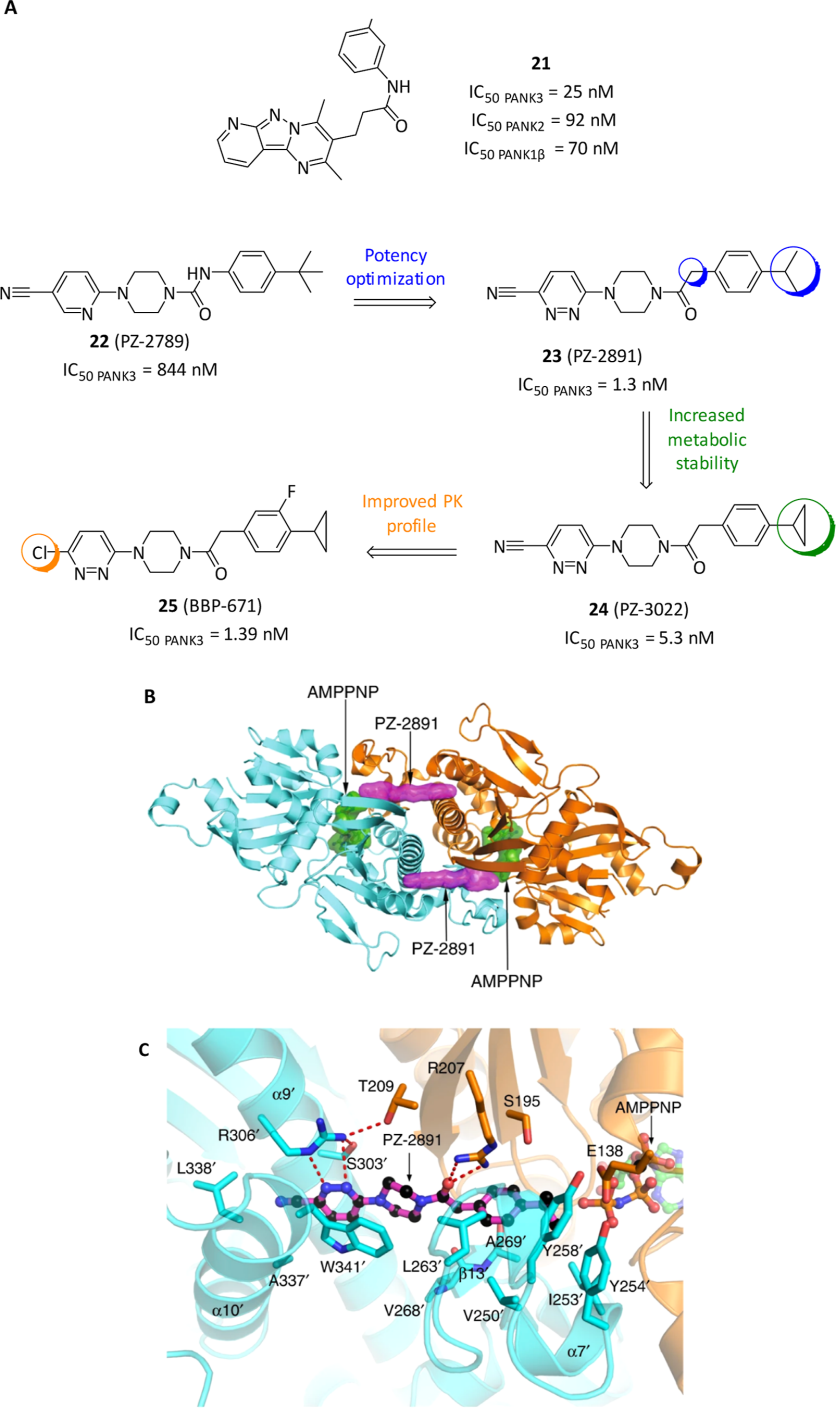
(A) Chemical structures and biological activities of PANK allosteric modulators. (B) Overview of the PANK3 dimer illustrating that **23** (PZ-2891) binds across the dimer interface. The two PANK3 protomers are colored cyan and gold, **23** is purple and AMPPNP is green. (C) Zoomed view of **23** bound across the dimer interface of the PANK3•AMPPNP•Mg^2+^complex illustrating the key hydrogen bonding interactions (dotted red lines) with both PANK3 protomers. Adapted from Sharma, L. K.; Subramanian, C.; Yun, M. K.; Frank, M. W.; White, S. W.; Rock, C. O.; Lee, R. E.; Jackowski, S. A therapeutic approach to pantothenate kinase associated neurodegeneration. *Nat. Commun.* **2018**, 9 (1), 4399. DOI: 10.1038/s41467-018-06703-2.^75^ Licensed under CC BY 4.0.

### p70 Ribosomal S6 Kinase

p70 ribosomal S6 kinase (p70S6), also called S6K1, is a Ser/Thr kinase belonging to the AGC protein kinase family like S6 kinases but differing from the other isoform S6K2. S6Ks play crucial role in regulation of protein synthesis and cell cycle through the phosphorylation/activation of the 40S ribosomal protein S6.^81^ Kinase activation requires phosphorylation at eight different sites, including four autoinhibitory pseudosubstrate sites, finely regulated by different upstream pathways such as mTOR, MAPK and PI3K.^82^ In CNS context, there are contradictory findings regarding its involvement in tau-mediated neurotoxic processes,^83^ while pivotal role in promoting oligodendrocyte differentiation during development and remyelination was credited to p70S6.^84^ Previously, therapeutic manipulation of p70S6 was suggested for potential treatment of age-related diseases.^85^ Furthermore, some p70S6 inhibitors were identified as autophagy inducers as well as favoring activation of important neuroprotective pathways such as Nrf2 or SOD1 aggregates degradation, thus prompting development of p70S6 modulators for neurodegenerative disorders.^86^

From a phenotypic screening aiming to identify small molecule able to promote neurogenesis emerged compound NNI-362 (**26**, Figure 8),^87^ which turned out as selective (i.e., in a panel of 151 kinases) p70S6 stimulator with an allosteric mechanism of action that also granted neuronal-selectivity. Particularly, cotreatment with CDK5 inhibitors resulted in amplified **26**-induced proliferation, thus suggesting activity at the same critical autoinhibitory allosteric site, Ser411, which is predominantly phosphorylated by CDK5 at the neuronal level. At 1 μM, **26** greatly promoted proliferation of neural progenitor cells with an increased ratio of mature neurons to total cells. In both aged and Down syndrome mice **26** at 10 mg/kg completely reversed the related cognitive deficits and increased neurons in the hippocampus, resulting safe up to 6 weeks of administration.^88^ In Phase IA clinical trial (NCT04074837) it appeared safe and well tolerated at doses up to 240 mg orally administered. Furthermore, during the same clinical trial the enrolled AD patients demonstrated reduced blood p-tau181, a critical AD biomarker.^89^ In AD and PD animal models **26** significantly increased neurons in hippocampus with memory impairment reversal and showed regeneration of dopaminergic neurons in the substantia nigra, respectively the two selective regions of neuron loss.^90^ Based on these premises, a phase II is planned for AD and PD patients.

**Figure 8:**
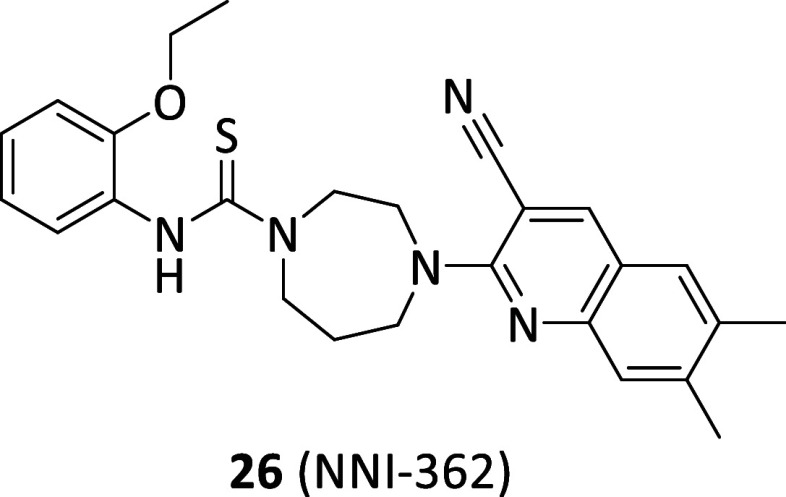
Chemical structure of the p70S6 allosteric modulator **26**.

## Kinases with Validated Neuroprotective Properties and the Respective Allosteric Modulators: Allosteric Strategies to Achieve Selectivity

Due to the substrate ubiquitously shared, the ATP site offers fewer and tricky possibilities to drive selectivity of action with ATP-competitive modulators; thus, targeting sites alternative to the ATP one emerges as a more suitable strategy in this respect. Loss of efficacy, adverse effects, or even toxicity are only few examples regarding the most common consequences of unwanted off-target effects which arise during preclinical drug development, while for what concerns pharmacological tools selectivity is intrinsically required by definition. Notably, over the years the development of allosteric kinase inhibitors enabled to reach kinome-wide, subtype or even mutant selectivity (e.g., as previously reported) and hereafter some examples in the context of neurodegeneration will be analyzed.

### Casein Kinase 1

Casein kinase (CK) is a term used to indicate three kinases that share the ability to phosphorylate casein in vitro, but only one of these, named GEF-CK (Golgi-enriched fraction CK) is literally a CK, while the other two denoted nowadays simply as CK1 and CK2, are pleiotropic enzymes having no functional relatedness whatsoever with casein.^91^ Particularly, CK1 is a ubiquitous kinase belonging to the Ser/Thr protein kinase family, which is constitutively active and self-regulated through phosphorylation at *C*-terminal. CK1 is a monomeric kinase characterized in seven different isoforms (i.e., α, β, γ1, γ2, γ3, δ and ε) and each of them features different activities, functions, subcellular localization, and biochemical properties. CK1 isoforms are highly expressed in cortex and striatum, regulating glutamatergic synaptic transmission, circadian rhythm and the modulation of Wnt/β-catenin and Hedgehog pathway during development.^92^ Its activity was linked to the development of different neurological disorders, particularly in tauopathies, where CK1 phosphorylates tau, a transactive response DNA binding protein of 43 kDa (TDP-43) and α-synuclein, besides orchestrating resulting pathogenesis as well Aβ neurotoxicity. For example, CK1α can be found in neurofibrillary tangles, while CK1δ is linked to granulovacuolar degeneration bodies and this altered function is related to the dysregulation of circadian rhythms in AD. In addition, elevated expression of CK1 isoform δ and ε has been found in AD and amyotrophic lateral sclerosis (ALS) postmortem brain tissues.^93,94^ Based on these premises, several CK1 inhibitors have been described, almost entirely ATP-competitive,^93^ and always relating to selectivity issue due to high structural analogy within binding sites of different isoforms.

Interestingly, two different allosteric modulators were disclosed as activators of specific CK1 isoforms with neuroprotective properties. In the first case, albeit with conflicting opinions, pyrvinium pamoate was first proposed as CK1α allosteric activator, resulting in Wnt signaling inhibition.^95^ Furthermore, the use of pyrvinium in reducing tauopathies has been recently patented (WO 2025/038296), although the exact mechanism of action must be fully clarified.

Regarding the second CK1 allosteric modulator, CK1γ2 was the isoform of interest. CK1γ2 is responsible for the phosphorylation of presenilin 1 (PS1), enzyme involved in γ-secretase complex, with resulting interference with the amyloidogenic cleavage and Aβ levels reduction.^96^ Particularly, during the amyloidogenic cleavage, the APP undergoes sequential proteolysis from β-secretase, forming C99 intermediate and the final γ-secretase generating different Aβ peptides. Therefore, the search for CK1γ2 activators resulted as a promising strategy to reduce the neurotoxic Aβ burden characteristic of several neurodegenerative diseases. Particularly, once the inhibitory effect on CK1γ2 activity was determined as the result of intramolecular autophosphorylation, research was devoted toward the identification of CK1γ2 autophosphorylation inhibitors. From HTS and following hit-to-lead optimization, compound **27** (CKR-49-17, Figure 9) emerged as one of the most promising CK1γ2 activators with proved ability to bind the target (*K*_d_ = 180 nM) and increase PS1 phosphorylation at Ser367 (EC_50_ = 60 μM).^97^ A small SAR campaign was reported: (i) small substituents (e.g., fluoro, nitro, cyano, trifluoromethoxy) were tolerated in position 6 of the benzothiazole core; (ii) a methylene bridge is preferred between benzamide and terminal heterocycles; and (iii) lateral free 4-aminopiperidine instead of morpholine increased activity. Computational investigations revealed the potential allosteric site of interaction close to the active site, where interactions with Asp128 drove the binding mode (Figure 9). Following binding and phosphorylation experiments with mutant D128A CK1γ2 confirmed a 3-fold reduction in binding affinity paired with an inability to trigger PS1 phosphorylation for **27**, confirming the proposed allosteric binding site. Finally, in cells, **27** decreased C99 levels (IC_50_ = 15 μM) without affecting APP load and with a resulting dose-dependent Aβ reduction.

**Figure 9:**
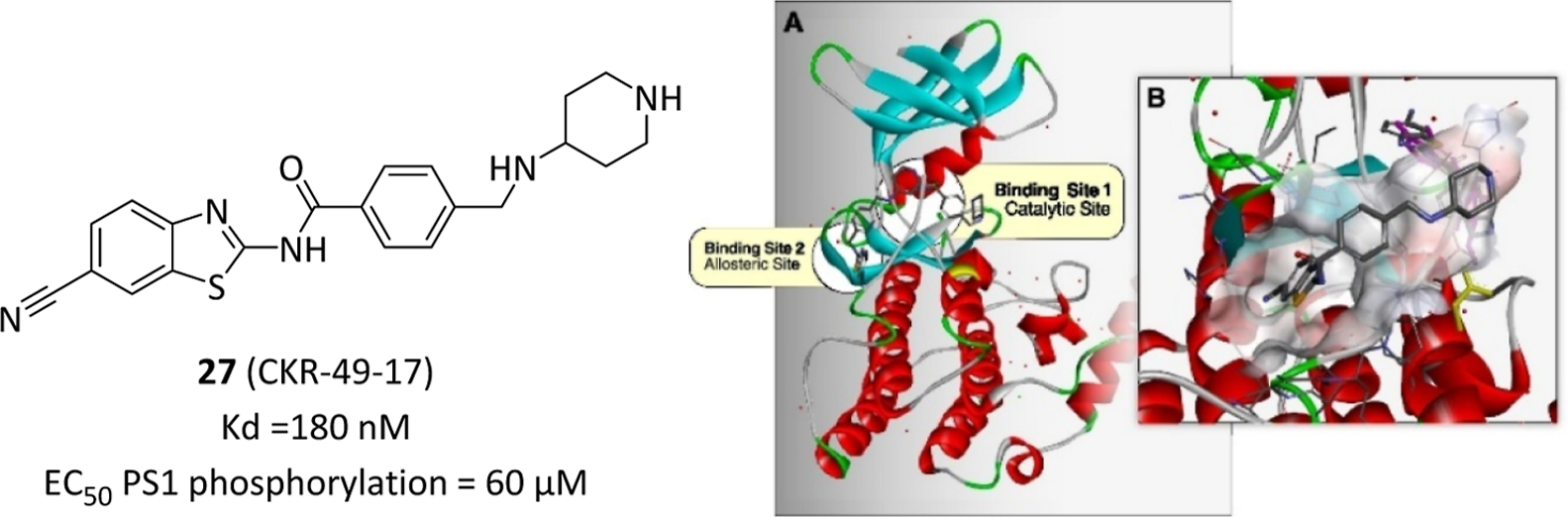
Chemical structure, biological activities of CK1γ2 allosteric activator **27** and allosteric binding site identification (A) with highlighted **27**’s binding mode (B). Reprinted with permission from Bustos, V. H.; Sunkari, Y. K.; Sinha, A.; Pulina, M.; Bispo, A.; Hopkins, M.; Lam, A.; Kriegsman, S. F.; Mui, E.; Chang, E.; et al. Rational Development of a Small-Molecule Activator of CK1γ2 That Decreases C99 and Beta-Amyloid Levels. *ACS Chem. Biol.* **2024**, 19 (1), 37–47. DOI: 10.1021/acschembio.3c00425.^97^ Copyright 2023 American Chemical Society.

### Phosphatidylinositol 5-Phosphate 4-Kinase

The phosphoinositide kinases regulate phosphatidylinositol signaling, which is involved in membrane trafficking, channel regulation, cell proliferation, and cell responses to stress and death. The several phosphorylated forms of phosphatidylinositol exert different cellular functions and are tiny regulated by a complex system of kinases and phosphatases.^98,99^ Particularly, phosphatidylinositol 5-phosphate 4-kinases (PI5P4Ks) tunes the conversion of phosphatidylinositol 5-monophosphate (PI5P) into phosphatidylinositol 4,5-bisphosphate (PI4,5P_2_) and consists of three isoforms (α, β and γ) with a very different intrinsic activity (α > β ≫ γ): while α and β isoforms have different physiological and pathological functions clarified, such as role in tumorigenesis,^98^ gene regulation and stress responses, PI5P4Kγ’s role is not completely understood.^100^ It is apparently expressed ubiquitously but at different rates among tissues, being especially high in kidney epithelial cells and in specific neurons of the brain. Given that they share a high degree of structural homology in the ATP-binding site, developing specific inhibitors has been critical to clarify the different functions of the isoforms. Therefore, allosteric modulators emerged as a potential approach to achieve specificity and finally elucidate their roles in pathophysiological conditions.

Compound **28** (NIH-12848, Figure 10A) was the first reported selective PI5P4Kγ modulator with potential for allosteric inhibition.^101^ To understand its exact binding site they performed IC_50_ determination in presence of both PI5P and ^32^P-γ-ATP (the substrate of the kinases): under these conditions with **28** at 100 μM the α isoform was not inhibited at all, β showed only small variations, and PI5P4Kγ was inhibited with an IC_50_ of 3.3 μM. A similar activity was also identified toward the mutant isoform PI5P4Kγ+ (IC_50_ = 1 μM) which is similar to the α one, suggesting that **28** interacts on a different site with respect to the ATP one. This was also in accordance with the results obtained testing the ATP rate conversion of PI5P4Kγ in the presence of different concentrations of **28**, which recalls the same ATPase activity in absence of inhibitor. Albeit there was not a conclusive competition experiment, further investigations suggested the lipid binding site as the putative region of interaction. Through an iterative optimization campaign compound **29** (Figure 10A) was later obtained with increased activity (IC_50_ = 0.63 μM) and solubility which lead to a cocrystal structure (PDB 7QIE). During SAR exploration, different ring systems were initially evaluated as a replacement of **28**’s thiophene ring, resulting in a preference for aromatic rings bearing a heteroatom in the *ortho* position. In the side benzene, only small lipophilic substituents were tolerated, almost exclusively in position 2 and preferably branched (e.g., isopropyl). In order to maximize the ligand efficiency and solubility, different central cores were also rated, identifying the pyrrolopyrimidine of **29** as the best balance in this respect. The crystal structure showed two different pockets for **29** that cannot be occupied simultaneously: the ATP binding site in chain B and the lipid binding pocket 18 Å far from the ATP site in other monomers (Figure 10B,C). In the latter conformation residues Gln378, Tyr379 and Asp380 from the activation loop occupy an inhibitory position in the ATP binding site closing it. Although the mechanism of interaction has been defined, the exact mechanism of inhibition remains to be elucidated, if may involve PI5P competition or pure allosteric inhibition which only induced detrimental conformational changes in the protein that disrupts interactions with distinct protein regions.

**Figure 10:**
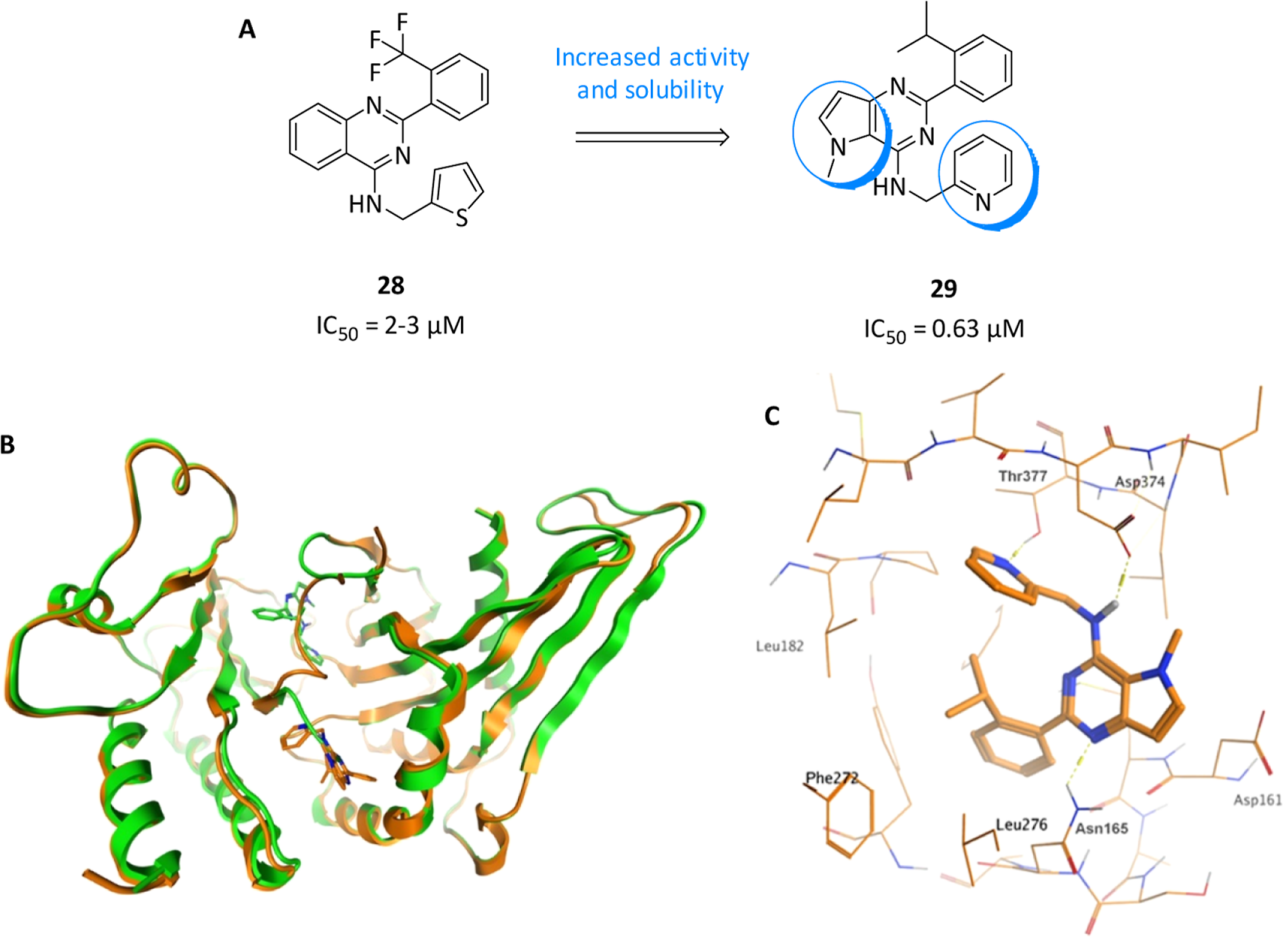
(A) Chemical structures and biological activities of PI5P4Kγ allosteric modulators. (B) The two binding sites for **29**: chain A in orange (**29** in allosteric binding pocket) and chain B in green (**29** in ATP site) superposed with **29** in the stick. (C) Allosteric binding pocket in chain A of the **29**-PI5P4Kγ complex with key interactions highlighted. Reprinted in part with permission from Boffey, H. K.; Rooney, T. P. C.; Willems, H. M. G.; Edwards, S.; Green, C.; Howard, T.; Ogg, D.; Romero, T.; Scott, D. E.; Winpenny, D.; et al. Development of Selective Phosphatidylinositol 5-Phosphate 4-Kinase γ Inhibitors with a Non-ATP-competitive, Allosteric Binding Mode. *J. Med. Chem.* **2022**, 65 (4), 3359–3370. DOI: 10.1021/acs.jmedchem.1c01819.^102^ Copyright 2022 American Chemical Society.

In parallel, another PI5P4Kγ putative allosteric inhibitor has been disclosed as potential strategy to tackle Huntington’s disease (HD) pathogenesis, relating for the first time this kinase as suitable target to mitigate huntingtin-related neurotoxicity.^103^ Particularly, **30** (NCT-504, Figure 11) stood out upon medicinal chemistry optimization of a series of 5-phenylthieno­[2,3-*d*]­pyrimidine compounds identified in a phenotypic HTS. After demonstrating a robust reduction of huntingtin (Htt) aggregates in cells, **30** was evaluated in a panel of 442 human kinases and turned out at 10 μM with only >65% PI5P4Kγ inhibition. In a further in vitro kinase assay, measured as phosphorylation of PIP5 by full length PI5P4Kγ, **30** showed an IC_50_ of 15.8 μM while it was inactive on α, β, and also PI5P4Kγ+. In addition, in the absence of PIP5 **30** was unable to inhibit the ATP-hydrolytic activity, suggesting an allosteric mechanism of action. As a confirmation of facts, at cellular level, **30** treatment led to the alteration of phosphatidylinositide levels, such as an enhanced PI5P, PI­(3,5)­P2 and PI3P expression in a dose- and PI5P4Kγ-dependent manner. Furthermore, it increased basal autophagy, whereas reduced the total amount of mHtt protein in human patient fibroblasts and aggregates in neurons in a similar fashion to PI5P4Kγ knock-down, offering overall promising prospects for an alternative strategy in HD drug discovery arsenal.

**Figure 11:**
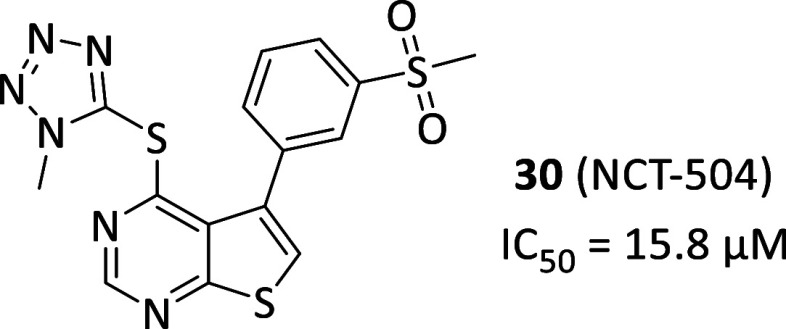
Chemical structure and biological activity of PI5P4Kγ allosteric inhibitor **30**.

### Dual-Specificity Tyrosine-Phosphorylation Regulated Kinase 1A

Dual-specificity tyrosine-phosphorylation regulated kinases (DYRKs) are ubiquitously expressed in the organism with signal transduction regulatory functions, which resulted in activation by autophosphorylation of tyrosine residues in the activation loop sequence. DYRK family consists of two different classes: one is composed by DYRK1A and DYRK1B, and the second by DYRK2, DYRK3 and DYRK4. All of them belong to Ser/Thr kinase family and share high structural homology, arising important selectivity issues.^104^ At physiological level, DYRK1A is involved in brain growth, neuronal development, and synaptic transmission, whereas it is highly overexpressed in the brain under neurodegenerative conditions as PD, AD, MS and Down Syndrome (DS).^105^ Regarding PD, DYRK1A is responsible for phosphorylating Ser131 of parkin, a protein involved in familiar PD, and α-synuclein, thus fostering its neurotoxicity and related dopaminergic dysfunction.^106^ A dysregulation of DYRK1A activity is also involved in AD pathogenesis: (i) it triggers amyloidogenic cleavage through APP phosphorylation; (ii) it interferes with both tau splicing and phosphorylation, besides favoring tau priming for GSK-3β phosphorylation; (iii) a toxic cycle is identified relating Aβ overload with resulting increased DYRK1A expression and consequent tau phosphorylation.^107^ Furthermore, given that DYRK1A gene is located in chromosome 21, its overexpression in DS contributes to DYRK1A-mediated β-amyloidosis with resulting brain deficit functions.^108^

Based on these premises, several competitive DYRK1A inhibitors are considered promising clinical candidates for neurodegenerative diseases treatments.^109^ At the same time, the current development of allosteric DYRK1A inhibitors mainly relies on epigallocatechin gallate (**31**, IC_50_ = 0.33 μM, Figure 12C) and its optimized derivatives.^110^ **31** resulted as noncompetitive DYRK1A inhibitor that in presence of Lys465 mutation shifted to competitive, suggesting a putative site for allosteric interaction.^111^ From computational investigations it seemed to locate in a flat pocket centered on Leu457, establishing pi–cation interaction between **31** and Lys222 and three hydrogen bonds with His424, Arg458 and Tyr462 (Figure 12A,B).^112^ To note, half of these residues are not conserved in DYRK1B, suggesting potential selectivity. Despite the interesting mechanism of action and promising experimental neuroprotective properties, it missed clinical translation due to poor bioavailability and lack of in vivo stability. To ameliorate its PK profile, a SAR campaign was conducted on the catechin scaffold by evaluating different substituents on the four-founding ring.^113^ In summary, trans conformation resulted preferred and almost all hydroxy functions verified as essential for optimal activity, with only fluorine insertion tolerated in *ortho* in B and D rings. Compounds **32** and **33** (Figure 12C) emerged as the most potent of the series but, once confirmed the noncompetitive mechanism of action, only **32** was evaluated in an inflammation murine model predictive for MS. While almost inactive after oral administration, by means of intranasal route at 15 mg/kg it resulted almost equipotent to the approved agent Fingolimod 1 mg/kg in terms of disease scores, pro-inflammatory cytokines production (e.g., TNFα, IFNγ, IL17) and lesions severity reduction. In pursuing adequate oral bioavailability, different OH-masking groups were later evaluated for the *meta*-position in D-ring.^114^ In this case, compound **34** with a methoxy group in that position gave the best results with an IC_50_ of 73 nM and acceptable selectivity over DYRK2 (Figure 12C). Even with these modifications, the pharmacokinetic profile remains poor, thus requiring a specific formulation with cyclodextrin and PEG-400 to achieve high drug concentration in dosing solutions for oral and intranasal route compared with the intravenous one. Regarding compound **34** the oral administration enabled a 16% bioavailability in plasma, while resulted almost complete via intranasal, differently from **31** and **33** which resulted almost null. Therefore, compound **34** was chosen to verify its in vivo efficacy to suppress neuroinflammation in LPS-induced inflammation and MPTP-induced PD mice models. In the first case, **34** orally administered at 30 mg/kg showed an important antinflammatory effect reducing TNFα accumulation in plasma and in brain paired to a decreased phosphorylated tau content in hippocampus. In the PD model, **34** at 25 mg/kg completely restored the impaired movement behavior with the oral-administered group generally showing superior efficacy compared to the intranasal one, probably because of the better brain/plasma ratio.

**Figure 12:**
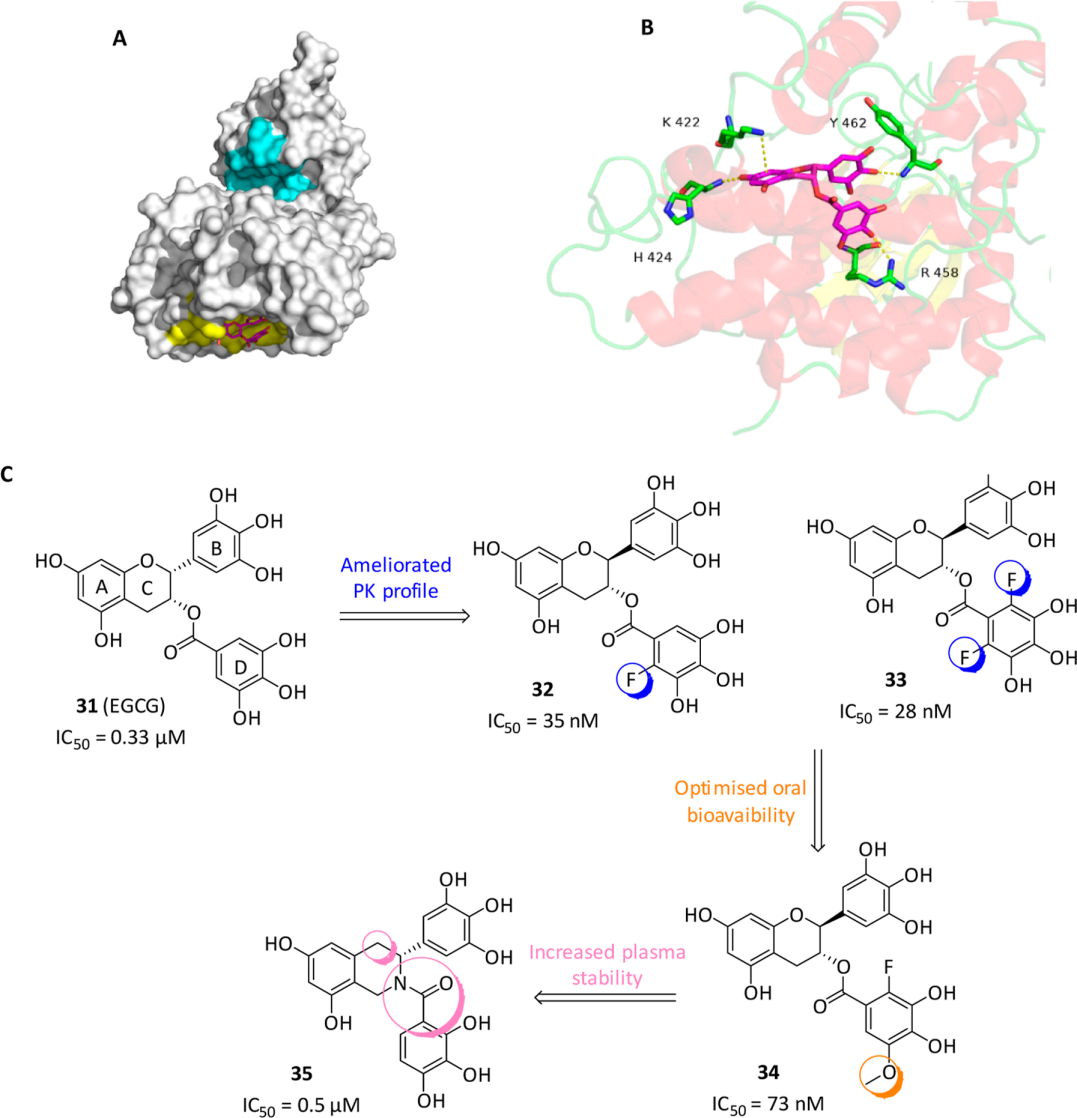
(A) The molecular surface of DYRK1A with highlighted catalytic site in cyan and putative **31**’s allosteric binding site in yellow. (B) Detailed interactions between DYRK1A and **31** (in magenta). Adapted from Gu, Y.; Moroy, G.; Paul, J. L.; Rebillat, A. S.; Dierssen, M.; de la Torre, R.; Cieuta-Walti, C.; Dairou, J.; Janel, N. Molecular Rescue of Dyrk1A Overexpression Alterations in Mice with Fontup. *Int. J. Mol. Sci.* **2020**, 21 (4). DOI: 10.3390/ijms21041404.^112^ Licensed under CC BY 4.0. (C) Chemical structures and biological activities of DYRK1A allosteric inhibitors.

More recently, to achieve the same aim (i.e., better in vivo stability), the ester was replaced with a shorter amide and the oxygen ring with a methylene group.^115^ The more similar EGCG analogue, compound **35** (IC_50_ = 0.5 μM, Figure 12C), resulted as the most potent derivative of the series, maintaining the noncompetitive mechanism of action and highlighting the important role of bis-3,4,5-hydroxyphenyl functions in this respect. Furthermore, within a small panel of 12 kinases, **35** observed a promising selectivity except for FYN and PI3K. The reported modifications greatly increased the plasma stability with respect to EGCG, whereas allowed almost the same brain bioavailability in mice. In a mouse model overexpressing DYRK1A **35** after 24h i.p. injection normalized ERK phosphorylation (i.e., DYRK1A target) similarly to WT. On the other hand, in a DS mouse model treated with **35** by gavage prenatally (5 days per week at 40 mg/kg) did not show almost any effect in terms of memory recovery.

### Receptor-Interacting Protein Kinase 1

Receptor-interacting protein kinases (RIPKs) are an heterogeneous family of Ser/Thr and tyrosine kinase-like kinases formed of seven members, albeit the last two (i.e., RIPK6 and RIPK7) are more structurally and functionally different to others and are better known as LRRK1 and LRRK2, respectively.^116^ RIPK1 is the RIPK founding member and represents a master regulator of the cellular fate in driving apoptosis or necroptosis efficiently through NF-κB activation in response to a broad set of inflammatory and pro-death stimuli TNF-orchestrated. It comprehends an N-terminal kinase domain regulating the activating autophosphorylation, an intermediate domain which contains RHIM sequence, and a C-terminal death domain (DD) which mediates homodimerization or heterodimerization with other DD-containing proteins as TNFR1, Fas and FADD to promote the activation of N-terminal domain.^117^

When cells are defective in activating apoptotic mediators (e.g., caspases), TNF-α stimulation promotes the activation of a secondary cytosolic “necrosome” complex formed by RIPK1, RIPK3 and mixed lineage kinase domain-like protein (MLKL) that interact through RHIM. This mechanism depends on the activation of RIPK1 which undergoes autophosphorylation on multiple residue: Ser14-15, Ser20, and Ser161-166 (the latter defined as biomarker for RIPK1 activation). The necrosome formation is related to a regulated necrotic mechanism known as necroptosis, which is defined as death-receptor-mediated caspase-independent cell death triggering inflammation. Otherwise, depending on cellular context and caspase activation, TNF-activated RIPK1 may control downstream mediators leading to apoptosis or increased inflammatory genes expression.^118^

In the CNS intrinsic apoptosis pathway is fundamental during development, while when mature neurons become more resistant to this regulatory process, thus favoring the extrinsic apoptosis, neuroinflammation and necroptosis take over. In this context, RIPK1 resulted a key effector orchestrating necroptosis and neuroinflammation implicated in several inflammatory and neurodegenerative diseases:^119^ (i) necroptosis markers as well as RIPK1 activation were found in AD postmortem brain;^120,121^ (ii) in MS cortical lesion brain samples were identified defective caspase-8 activation paired to activation of necroptosis markers (i.e., RIPK1, RIPK3 and MLKL);^122^ (iii) in ALS patients RIPK1 mediated axonal degeneration by inducing necroptosis and inflammation;^123^ (iv) increased expression of RIPK1 and RIPK3 related to progressive neuronal loss was found in patients affected by Niemann-Pick type C1 disease, a neurodegenerative lysosomal storage disorder.^124^ Furthermore, in all these cases, and not only, RIPK1 inhibition proved to have strong therapeutic potential for neurodegenerative disorders treatment.

The first RIPK1 inhibitors emerged from a phenotypic screening, demonstrating for the first time the existence of an alternative nonapoptotic cell death pathway triggered by a class of inhibitors called necrostatins. It is important to clarify how the development and characterization of necrostatins resulted indispensable for developing our current understanding of necroptosis biology and in parallel uncovering the role of the RIPK family at the pathophysiological level. Among the different classes of compounds developed, only necrostatin-1 family reached valid potencies and, particularly, necrostatin-1 (Nec-1, **36**, Figure 13A) was first identified able to selectively block the necroptotic death in vitro (EC_50_ = 494 nM) and in vivo without affecting apoptosis or autophagy with resulting neuroprotective properties.^125^ To explain this, **36** was later identified as specific type III RIPK1 inhibitor, albeit resulted ATP-competitive in kinetic assays, locating into the allosteric site behind the RIPK1 ATP-binding pocket, bearing RIPK1 inhibition rate perfectly matching with antinecroptotic potency in cells.^126^ Furthermore, the selective modulation of RIPK1 and TNF-induced necrotic cell death by necrostatins allow to selectively act on TNFR1 signaling, involved in CNS diseases, without affecting TNFR2 which instead mediates neural regeneration.^118^ Particularly, **36** inhibits necroptosis triggering the dimerization of the RIP1 kinase domain and modulating RIPK’s downstream messengers. Indeed, RIPK1 has an autophosphorylation site at Ser161 that can be important for its regulation because the activation segment can occlude the catalytic cleft of the kinase. As a confirmation of facts, the destabilization of the “closed” T-loop inhibits the kinase, consistent with the evidence that RIPK1 activity and **36**-mediated inhibition decrease when Ser161 is mutated.^126^

**Figure 13:**
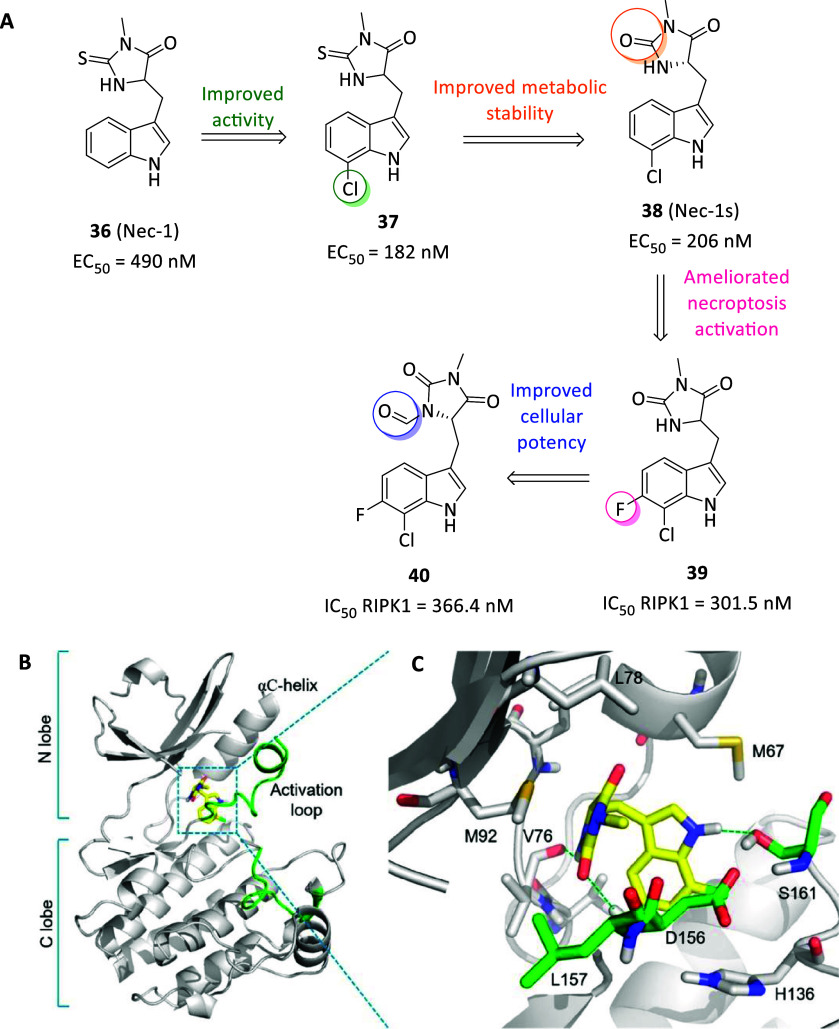
(A) Chemical structures and biological activities of RIPK1 allosteric inhibitors based on the necrostatin scaffold. EC_50_ measured as antinecroptotic activity. (B) Front view and (C) zoomed view at the binding site of RIPK1 and **38**. Reprinted with permission from Zhuang, C.; Chen, F. Small-Molecule Inhibitors of Necroptosis: Current Status and Perspectives. *J. Med. Chem.* **2020**, 63 (4), 1490–1510. DOI: 10.1021/acs.jmedchem.9b01317.^138^ Copyright 2019 American Chemical Society.

Subsequent SAR campaigns revealed limited development possibilities with only few modifications tolerated such as 7-chlorine insertion (**37**, EC_50_ = 182 nM, Figure 13A) or sulfur-to-oxygen replacement in the hydantoin core (Nec-1s, **38**, EC_50_ = 206 nM, Figure 13A) which led to increased antinecrotic activity and metabolic stability. Despite several beneficial effects, **36** demonstrated in vivo instability, toxicity at high concentrations and off-target effects (i.e., inhibition of indoleamine 2,3-dioxygenase), while **38** demonstrated to overcome these liabilities.^127,128^

Cocrystal structure with **38** (PDB 4ITH) revealed the exact binding site in a hydrophobic pocket between the N- and C-lobes, in close proximity of the activation loop, thus locking RIPK1 in an inactive conformation (i.e., DLG-out conformation and rotated αC-helix) through H-bond interaction with highly conserved residues (Figure 13B,C).^129^ This peculiar allosteric pocket and necrostatin selectivity is possible only thanks to the increased flexibility of the DLG motif in RIPK1 compared to other kinases.^130^

However, also **38** highlighted a suboptimal PK profile, demonstrating low exposure and high clearance,^127^ but still remains with **36** the most studied RIPK1 inhibitor. Here it is reported a nonexhaustive list of their in vivo evaluation in CNS context:^119,124,131^ (i) in MS mice models **38** ameliorated disease pathology, improved animal behavior, and attenuated neuroinflammation and oligodendrocyte death;^122^ (ii) in ALS mice model **38** blocked oligodendrocyte death,^123^ microglial inflammation and axonal degeneration, while **36** blocked motor neuron loss in patient-derived cell cultures;^132^ (iii) in PD animal models **38** prevented dopaminergic neuronal loss,^133^ while Nec-1 slowed dopaminergic degeneration, slowed astrocytic activation and improved motor/behavioral functions;^134^ (iv) in AD mice **38** attenuated the behavioral deficits and mitigated amyloid plaque-associated microglia and proinflammatory cytokines burden.^121^

More recently, further optimization on **36** core led to the disclosure of compound **39** (Figure 13A) which highlighted 8-fold more potent RIPK1 inhibition at cellular level in comparison to **38** with resulting amplified antinecroptotic activity.^135^ Furthermore, the introduction of an amide function (**40**, ZJU-37, Figure 13A) boosted RIPK1 inhibition (IC_50_ = 366.4 nM vs 1139 nM of **38**) and protective potency on cellular necroptosis (EC_50_ = 185.2 nM vs 1089 nM of **38**). In this case, **40** promoted OPC proliferation within the demyelination lesion and enhanced remyelination in a demyelinating mouse model in RIPK1-dependent manner,^136^ prompting an interesting role for RIPK1 inhibitors as potential therapeutics for these pathologies. Due to the outstanding results achieved within **36** family, several other necrostatin analogues or **36**-derived hybrids were developed among the years revealing notable antinecroptosis activities through allosteric RIPK1 inhibition, but always lacking any potential clinical translation.^130,137^

Another successful story of RIPK1 inhibitors started from the identification of benzoxazepinone **41** (GSK481, IC_50_ = 1.3 nM) from the DNA-encoded small-molecules library screening performed at GSK (Figure 14A). It showed a remarkable selectivity among the kinome plus species selectivity for primate vs nonprimate RIPK1, maintaining the same necrostatin mechanism (i.e., ATP-competitive and interacting with the kinase as type III inhibitor).^139^ Particularly, the benzyl group of **41** lied in the same allosteric lipophilic pocket of **38**, while the benzoxazepine ring occupied the same space of α-phosphate with a consequent C-helix shift and a more ordered activation loop in respect to **38** bound. The acceptable PK profile of **41** was further optimized in terms of lipophilicity, solubility and oral exposure in preclinical species, achieving compound **42** (GSK2982772, Figure 14A) as potential benzoxazepinone clinical candidate (IC_50_ = 1.0 nM).^140^ In cellular systems, compound **42** blocked TNF-downstream signals and mitigated cytokines production (i.e., IL-1β and IL-6) in the nanomolar range, thus fostering its clinical evaluation until phase IIa but only for peripheral pathologies (e.g., psoriasis, rheumatoid arthritis, and ulcerative colitis) due to low brain penetration. As example, a follow-up difluorinated benzazepinone analogue (GSK3145095) reached phase II in clinical trial for potential treatment of pancreatic cancer (NCT03681951).^141^ In pursuing benzoxazepinone analogues more active at central level, a two-step optimization procedure was developed at Takeda:^142^ (i) hybrization approach of **42** and **43**, an HTS-identified RIPK1 inhibitor locating in the usual allosteric pocket, to ameliorate the overall PK profile (compound **44**, Figure 14A); (ii) brain exposure enhancement. From this workflow derived compound **45** (Figure 14A) with excellent potency, selectivity and satisfying brain permeability, showcasing a type III binding mode superimposable with the same of **42** in addition to a H-bonding network between Asp56 and N1 and carbonyl of central bicyclic core, cyano pointing toward the solvent and chlorine interacting with Met67 (Figure 14B). After confirming target association with RIPK1 in mouse brain tissues and remarkable necroptosis suppression at the cellular level (IC_50_ = 2.0 nM in HT-29 cells and 15 nM in L929 cells), compound **45** was further evaluated in experimental autoimmune encephalomyelitis (EAE) MS mouse models. Orally administered at 20 mg/kg/day **45** significantly lowered clinical symptoms development of EAE, representing one of the most promising RIPK1 inhibitors with neuroprotective potential. Plenty of other allosteric inhibitors based on this scaffold were reported among recent years with floating potencies or metabolic liabilities, mainly directed for peripheral inflammatory diseases.^143^

**Figure 14:**
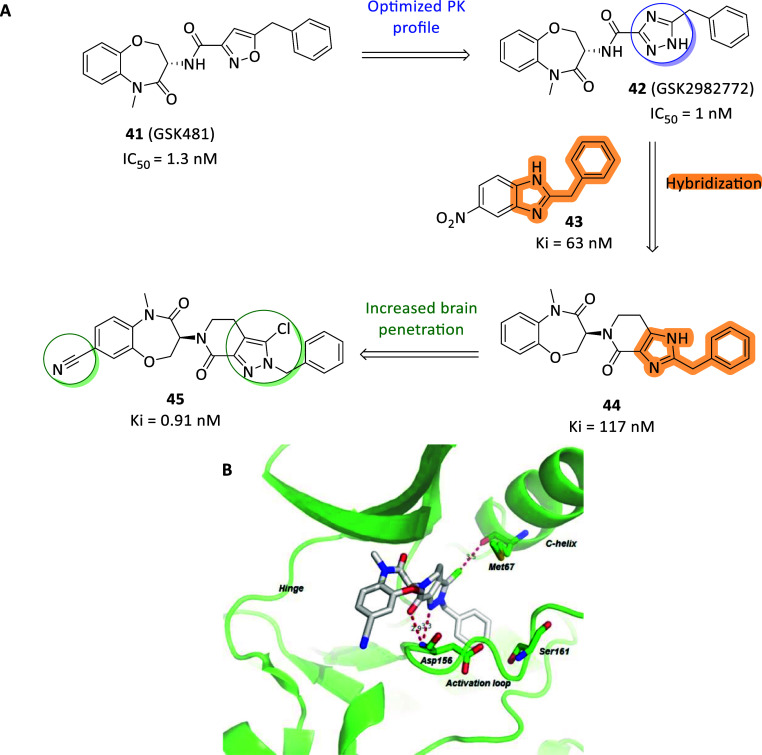
(A) Chemical structures and biological activities of RIPK1 allosteric inhibitors based on a benzoxazepinone scaffold. (B) Crystal structure of compound **45** and RIPK1. Reprinted with permission from Yoshikawa, M.; Saitoh, M.; Katoh, T.; Seki, T.; Bigi, S. V.; Shimizu, Y.; Ishii, T.; Okai, T.; Kuno, M.; Hattori, H.; et al. Discovery of 7-Oxo-2,4,5,7-tetrahydro-6 H-pyrazolo­[3,4- c]­pyridine Derivatives as Potent, Orally Available, and Brain-Penetrating Receptor Interacting Protein 1 (RIP1) Kinase Inhibitors: Analysis of Structure–Kinetic Relationships. *J. Med. Chem.* **2018**, 61 (6), 2384–2409. DOI: 10.1021/acs.jmedchem.7b01647.^142^ Copyright 2018 American Chemical Society.

From another GSK HTS emerged the dihydropyrazole GSK963 (**46**, IC_50_ = 8 nM, Figure 15) as selective RIP1K inhibitor demonstrating potent and specific antinecroptotic activity in cells and promising in vivo effects.^144^ Further efforts were devoted first toward increased potencies (DHP76, **47**, IC_50_ = 1 nM in vitro and 4.0 nM in cells) and second to ameliorate PK profile until obtaining DHP77 (**48**) representing the better compromise between good biological activities (IC_50_ = 20 nM in vitro and 63 nM in cells) and low clearance, good exposure, and good oral bioavailability (Figure 15).^145^ Even in this case, **48** bound to the lipophilic allosteric region in the back of the ATP pocket establishing essential H-bond between the pyrazole carbonyl and Asp156 backbone. **47** was later tested in EAE or retinitis pigmentosa mice models, due to a less primate species-specific RIP1K inhibition. In this case, orally administered at 96 mg/kg/day, **47** demonstrated neuroprotection with an induced delay in disease onset and reduced clinical severity, while protected retinal cell functions and survival at 100 mg/kg/day.

**Figure 15:**
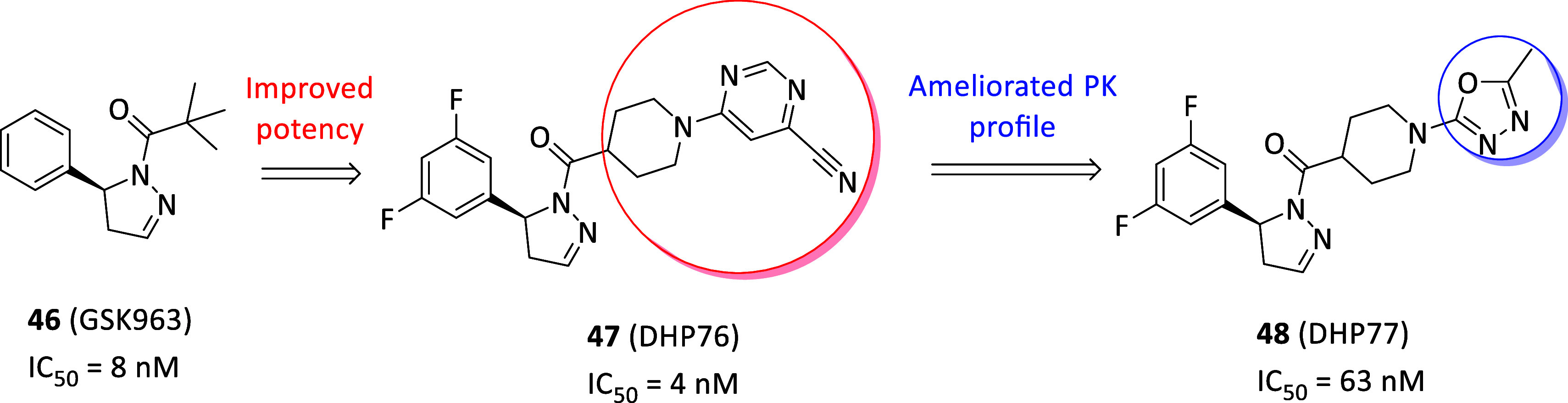
Chemical structures and biological activities of RIPK1 allosteric inhibitors based on a dihydropyrazole scaffold.

Several other different scaffolds had proven suitable for developing type III RIPK1 inhibitors, mostly anticancer or anti-inflammatory agents peripherally restricted without registered brain interest to date.^146^ One example is RIPA-56 (**49**, IC_50_ = 13 nM, Figure 16), potent selective and metabolically stable inhibitor with proven efficacy in reducing inflammation and glutamate-induced excitotoxicity in different mice models.^147^

**Figure 16:**
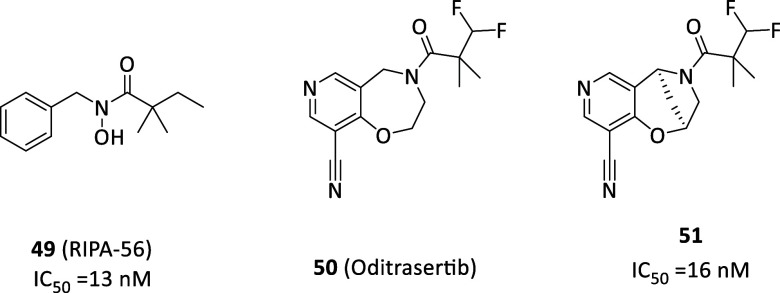
Chemical structures and biological activities of other RIPK1 allosteric inhibitors.

A special mention is deserved to the allosteric RIPK1 inhibitor pipeline carried out at Denali Therapeutics (WO2018213632A1). In collaboration with Sanofi two different compounds reached advanced clinical trials for neurodegenerative diseases treatment.^148^ Particularly, DNL747 (later called SAR443060) represented the first derivative of the series reaching Phase Ib for AD (NCT03757325) and ALS (NCT03757351), albeit subsequently discontinued for toxicity issues.^149^ A follow-up derivative called DNL788 (**50**, also known as SAR443820 or Oditrasertib, Figure 16) was further advanced into Phase II for ALS (NCT05237284) and MS (NCT05630547), but unfortunately recently discontinued because missing primary end points.^150^ To note, a similar bridged benzoxazepine **51** (IC_50_ = 16 nM, Figure 16) was newly disclosed by Merck, revealing great potency paired with promising PK and CNS permeability properties, thus deserving further investigations.^151^

The high interest for this class of compounds is corroborated by the vastness of patent literature in this field,^152^ to whom the interested reader is directed. Moreover, the entire journey on RIPK1 allosteric inhibition from necrostatin discovery until clinical trials of benzoxazepines highlighted the importance of a proper structural characterization of the target of interest in pursuing allosteric drug development as well as the aid (or not) given from specific “structural peculiarities” in leveraging allosteric pockets for therapeutic purposes.

### LIM Kinase

LIMK1 and LIMK2 are LIM kinases belonging to the tyrosine kinase-like family (TKL) which act as dual specificity kinases recognizing both Ser/Thr and Tyr-containing substrates. The structural homology in LIMK1 and LIMK2 is high (i.e., overall sequence conservation >50%), because they share the same domain organization with *N*-terminal LIM domains, a PDZ domain and a Pro/Ser-rich region before the *C*-terminal domain.^153^ Usually, LIMKs work as downstream effectors of the Rho GTPase family signaling pathways (i.e., Rho, Rac and Cdc42) that are known to activate LIMKs, via the corresponding kinases (e.g., ROCK, PAK, MRCKα), through phosphorylation at Thr508 for LIMK1 or Thr505 for LIMK2.^154^ Based on these upstream signals, LIMK1/2 regulate actin and microtubule dynamics, thus modulating several pivotal cellular processes such as cell cycle, survival and neuronal development.^155^ Particularly, through phosphorylation and inactivation of cofilin protein family they regulate the ratio between globular (G) and filamentous (F), while their dysregulation led to F-actin accumulation and consequent abnormal synaptic and dendritic spine morphology.^154^ Furthermore, LIMK impaired activity has been detected in several CNS disorders such as AD, PD, MS, and FXS, thereby indicating its inhibition as potential treatment for these diseases.^156^ Particularly, increased *p*-LIMK1 or *p*-cofilin in postmortem brain tissue as well as abnormally high density of dendritic spines in cortical neurons characterized FXS patients, leading to defects in synaptic plasticity which underlie the clinical symptoms, while the pharmacological inhibition of LIMK ameliorates the aberrant spine development in diseased animal model.^157^

In 2014 a first HTS reported a new LIMK2 inhibitor bearing a sulfonamide moiety active in the micromolar range (**52**, Figure 17A).^158^ Reversing the *S*-thiophenylsulfonamide to the *N*-phenylsulfamoyl side group allowed a 800-fold increase in potency, achieving a potent selective nanomolar allosteric LIMK2 inhibitor (compound **53**, Figure 17A). Starting from **53** a preliminary SAR campaign was conducted without achieving meaningful improvements. For solubility reasons, compound **54** (IC_50_ = 92 nM, Figure 17A) was chosen for X-ray crystallography, confirming type III allosteric binding for this class of compounds. Particularly, it locates in the hydrophobic pocket formed when the DFG residues within the activation loop are in the DFG-out conformation. The binding mode showed pivotal interactions between the carbonyl of the amide with NH’s backbone of Asp469 and an H-bond between the sulfonamide oxygen with Arg474, while the attached phenyl group established some hydrophobic interactions and the benzylamide moiety pointed out to the solvent front.^158^

**Figure 17:**
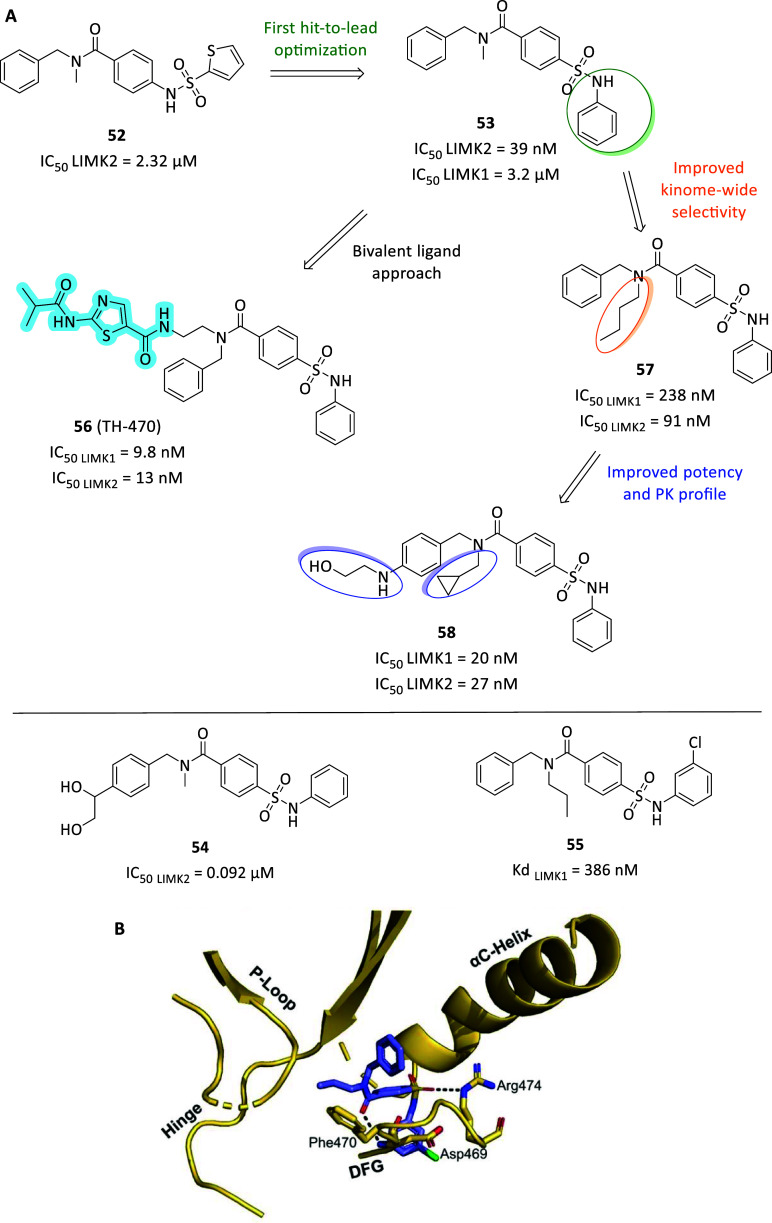
(A) Chemical structures and biological activities of LIMK allosteric inhibitors (1). In bivalent ligand **56** it is highlighted in pale blue the hinge binder motif. (B) Cocrystal structure of compound **55** (in violet) bound to LIMK2 (yellow). Reprinted in part with permission from Hanke, T.; Mathea, S.; Woortman, J.; Salah, E.; Berger, B. T.; Tumber, A.; Kashima, R.; Hata, A.; Kuster, B.; Müller, S.; et al. Development and Characterization of Type I, Type II, and Type III LIM-Kinase Chemical Probes. *J. Med. Chem.* **2022**, 65 (19), 13264–13287. DOI: 10.1021/acs.jmedchem.2c01106.^159^ Copyright 2022 American Chemical Society.

A wider SAR exploration on the same core was later conducted, confirming the precedent clues: (i) the benzyl moiety on the amide side remained the best substituent; (ii) small modifications were tolerated on the sulfonamide-attached ring; (iii) the amide had to be tertiary, with the methyl substitution that can be elongated maintaining the activity.^159^ In this case, the cocrystal structure of compound **55** (Figure 17B), chosen for suggested selectivity from DSF studies but confirmed dual submicromolar binder from ITC (i.e., LIMK1 *K*_d_ = 386 nM), with LIMK2 confirmed the type III allosteric binding in αC- and DFG-out conformation. In particular, the αC-helix, the P-loop and the DFG motif were greatly rearranged, confirming their high flexibility in unphosphorylated LIMK. Otherwise, through the elongation of the alkyl chain a rearrangement of this flexible regions occurred opening the access to the ATP-binding site. The latter observation allowed the construction of type II inhibitors merging the phenylsulfamoyl moiety of identified type III inhibitors and the 2-aminothiazole hinge-binder fragment, achieving compound **56** (Figure 17A) with exquisite dual potency and able to simultaneously interact with the P-loop, DFG motif, and ATP-binding site. Compound **57** (IC_50_ LIMK1 = 238 nM, IC_50_ LIMK2 = 91 nM, Figure 17A), analogue of **53** bearing a better pharmacokinetic profile, highlighted an outstanding kinome and cellular phosphorylation response selectivity profile with respect to LIMK type I and II inhibitors, remarking the potential of allosteric inhibition in this respect. Finally, in a FXS cellular model compound **57** inhibited neurite outgrowth in a dose-dependent manner more efficiently than the ATP-competitive counterpart and dose-dependently reduced *p*-cofilin in human cortical neurons of FXS patients.^159^

Unfortunately, compound **57** suffered of poor aqueous solubility and rapid microsomal turnover and, to overcome these PK issues, an optimized allosteric series has been recently disclosed.^160^ In this regard, in the alkyl chain switching from *N*-butyl to *N*-methylene cyclopropyl increased the potency, while introducing substituents in the *para* position of the benzyl ring improved the metabolic stability. Particularly, compound **58** (Figure 17A) emerged with excellent selective LIMK1/2 inhibitory potency, significantly improved cell permeability, lowered drug efflux and optimal in vivo PK profile. Computational investigations into LIMK1 homology structure located the cyclopropyl moiety in the hydrophobic region lined with Val366, the hydrophobic chain of Lys368, Leu397, Thr413 and Phe479, while appended NH of the ethanolamine interacted with Glu369 and terminal hydroxyl H-bonded with Glu369 and Ile371 besides being free to rotate and able to interact with environmental water. No adverse clinical signs were observed after 28 days of treatment with **57** (dosed at 30 mg/kg/day, ip) in mice, hence making it appropriate for potential chronic treatment. In hippocampal slices of FXS mice **58** at 3 μM decreased *p*-cofilin levels, while in similar in vivo model the perfusion of **58** (3 μM) produced a significant enhancement of hippocampal LTP, thus confirming its efficacy both ex vivo and in vivo for potential FXS treatment.

More recently, two novel chemical families of LIMK inhibitors were reported. First, due to the similar αC-out and DFG-out conformation in binding modes of benzoxazepinone-based RIPK1 inhibitors and **55** in LIMK, benzoxazepinones were repurposed as LIMK allosteric inhibitors.^161^ After screening of a small library in this family, compound **59** was identified as a potent LIMK1/2 ligand (Figure 18). Cocrystal structure of **59** with LIMK1 confirmed to occupy the same housing within the back pocket of the catalytic domain created by the αC-out and DFG-out conformation of other LIMK allosteric inhibitors, mainly driven by aromatic hydrophobic interactions in this case. Furthermore, besides the original target RIPK1, **59** revealed great selectivity for LIMK1/2 with a cellular on target activity in the nanomolar range (LIMK1 EC_50_ = 51 nM, LIMK2 EC_50_ = 40 nM). Albeit low oral bioavailability, but the good metabolic stability paired to no cytotoxicity and almost nullified cofilin phosphorylation at 1 μM, prompted compound **59** for future investigations regarding its therapeutic potential.^162^ From **59**’s crystal structure and following a structure-based drug design, the tetrahydropyrazolopyridinone **60** (Figure 18) was recently disclosed as the most selective LIMK1/2 allosteric inhibitor bearing promising in vivo drug-like properties, thus deserving prospects for future therapeutic evaluation.^163^

**Figure 18:**
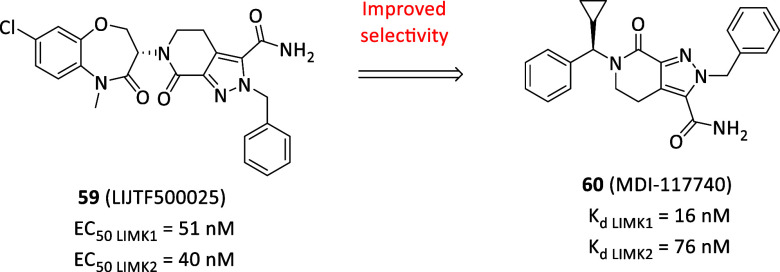
Chemical structures and biological activities of LIMK allosteric inhibitors (2).

Second, multiple virtual screening campaigns were recently reported to identify new allosteric LIMK2 inhibitors.^164^ Among these, particularly, based on **56**’s binding mode,^159^ several tetrapeptides were virtually screened and ranked for potential selectivity, affinity and PK properties.^165^ In particular, Tyr-Phe-Tyr-Trp (**61**) for LIMK1 and Trp-Phe-Val-Trp (**62**) for LIMK2 resulted as putative potent and specific allosteric inhibitors occupying the same back pocket of the above-mentioned kinases, deserving prospective in vitro evaluation.

## Kinases with Potential Interest for Neuroprotective Activity and the Respective Allosteric Modulators

### Casein Kinase 2: the Importance in Reaching Subtype Selectivity

Differing from its original relative CK1, CK2 is a constitutively active Ser/Thr kinase which exists as a heterotetrametric holoenzyme consisting of two catalytic subunits, α and α′, and a dimer of regulatory subunits β.^166^ The catalytic forms CK2α and CK2α′ are both active in absence of CK2β, but this latter confers specificity toward the substrate.^167^ CK2 is a ubiquitous kinase which, by exploiting ATP or GTP as phosphoryl donors, intervenes as versatile controller of many fundamental biological processes such as cell growth and proliferation.^168^ Its expression pattern in several cancer tissues paired with the antitumoral effects arising from its inhibition made CK2 an interesting target in anticancer therapy. To date, an ATP-competitive CK2 inhibitor (i.e., Silmitasertib), CK2α-binding, was granted as an orphan drug by the FDA in 2017 and is in advanced clinical trials for treatment of several types of cancer.^169^

In the brain CK2 acts as regulator of different neuronal functions depending on the individual subunits which showed distinct expression pattern and substrate targets.^170,171^ Generally, besides brain-wide pathological overexpression, CK2 modulates the phosphorylating status of multiple target proteins involved in neurodegenerative disorders (e.g., presenilin, huntingtin, α-synuclein, tau), thus accounting for an important role in disease development.^93,168^ Furthermore, different CK2 subunits were stained colocalized within pathological inclusions such as Lewy bodies or NFT.^92^ In AD and PD CK2 emerged as important driver in neurotoxicity processes, suggesting putative therapeutic perspective arising from its inhibition.^171,172^ In HD only CK2α′ resulted induced and involved in neuroinflammatory and neurodegenerative processes, while CK2 inhibition provided detrimental effects in this respect, thus requiring specific inhibitory activity to achieve therapeutic purposes.^173^

Several CK2 inhibitors have been investigated among the years as potential neuroprotective agents with poor performance,^92^ while any allosteric modulators were evaluated in this respect albeit comprehensive studies on CK2 allosteric pockets identification and characterization.^174,175^ Particularly, due to the high structural homology among subunits paired to their different biological roles, the development of CK2 allosteric modulators may be a valuable tool to finely dissect their functions at the cellular level. First, an exosite on CK2α was suggested as putative binding site for a class of polyoxometalates (POMs), inorganic compounds which resulted as potent and selective noncompetitive CK2 inhibitors.^176^ Noteworthy, different POMs were later characterized as substrate-competitive CK2 inhibitors.^177^

To date, four different CK2 allosteric sites have been disclosed (Figure 19A): three proximal (i.e., pockets 1, 2 and 3) to the ATP-binding site and one distal (i.e., type IV allosteric pocket). This last one, called “D” pocket, was recently identified in the N-terminus of CK2α′, bearing prospects for selective targeting thanks to different constituting residues in respect to CK2α.^178^ To detect novel D pocket binders, an HTS on a commercial library of allosteric kinase inhibitor-like compounds was carried out. In this way, the two selective CK2α′ inhibitors discovered confirmed the HD therapeutic potential within this class of compounds, by reducing HTT aggregation at the cellular level in the micromolar range. Unfortunately, competition experiments revealed that the two identified inhibitors acted as ATP-competitive ones, thus recalling future chemical explorations to validate this potential type IV allosteric pocket.^178^

**Figure 19:**
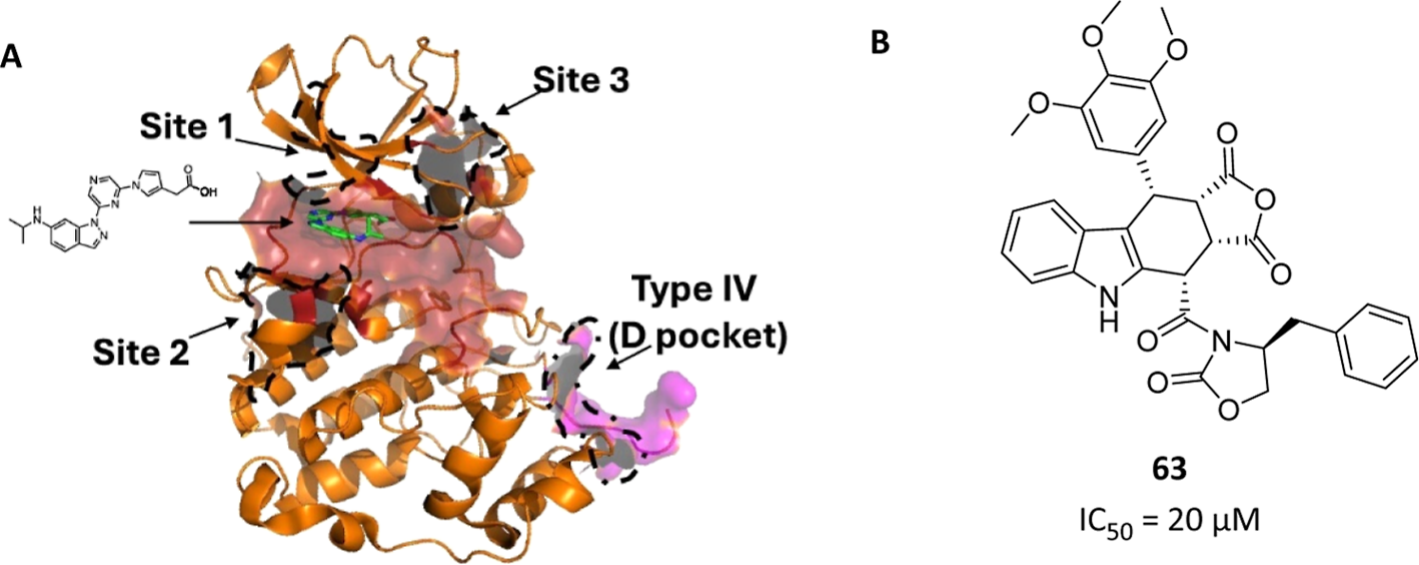
(A) CK2α′ protein structure with an ATP-competitive inhibitor highlighting the active site and the four allosteric pockets. Reprinted in part with permission from Mudaliar, D.; Mansky, R. H.; White, A.; Baudhuin, G.; Hawkinson, J.; Wong, H.; Walters, M. A.; Gomez-Pastor, R. Discovery of a CK2α′-Biased ATP-Competitive Inhibitor from a High-Throughput Screen of an Allosteric-Inhibitor-Like Compound Library. *ACS Chem. Neurosci.* **2024**, 15 (15), 2703–2718. DOI: 10.1021/acschemneuro.4c00062.^178^ Copyright 2024 American Chemical Society. (B) Chemical structure and biological activity of CK2 allosteric inhibitor targeting allosteric pocket 1.

Based on the key observation that dynamic association of CK2 subunits contribute to kinase activity regulation, allosteric site 1 was first identified and located at the interface between α and β subunits (i.e., Tyr39, Val67, Val112 and Val101).^179^ This hypothesis was further confirmed by the inhibitory potency and antagonist effect on CK2 complex assembly of a series of structure-based designed CK2β-derived cyclic peptides.^180^ A final validation for this CK2β binding pocket in CK2α as amenable allosteric site came from the podophyllotoxineindolo derivative **63** (CK2α IC_50_ = 20 μM, Figure 19B) bearing a noncompetitive CK2α inhibitory mechanism and resulting from a CK2α/CK2β interaction HTS.^181^ In this context, other allosteric binders were reported affecting the CK2β-dependent substrates phosphorylation but without maintaining CK2α inhibitory properties.^174^

In a fragment screening targeting α/β interface on the catalytic CK2α subunit the site 2 (Figure 19A) was first revealed as a new druggable pocket near the ATP-binding site, locating behind the αD helix and therefore named αD site.^182^ This pocket is formed by the movement of the flexible αD helix, which opens up a deep hydrophobic cavity adjacent to the ATP site. In CK2α the αD helix is more flexible and unusually adaptable than in other kinases and can adopt multiple positions: the closed conformation, the partially opened conformation, and the inactive conformation where there is Leu134 that fills the αD pocket and prevent the interaction with ATP or GTP through a distortion of the hinge region. Among the multiple binding sites, when 3,4-dichlorophenethylamine (**64**, Figure 20) bound in αD pocket displaced Tyr125 from its normal position and Met225 rotated opening the bottom of this pocket. Further affinity and selectivity optimization led to compound **65** (*K*_d_ = 270 μM, Figure 20) with the terminal phenyl group buried deep in the hydrophobic αD site. However, these compounds did not show any CK2α inhibitory activities; therefore, to achieve efficient inhibitors these two fragments were linked to ATP site warheads. After iterative linker optimization process, from **65**’s fragment derived the bivalent ligand CAM4066 (**66**, CK2 *K*_d_ = 0.31 μM, Figure 20) which, occupying both αD and ATP-binding pocket, inhibited kinase activity of both CK2α (IC_50_ = 0.37 μM) and CK2 complex (IC_50_ = 0.67 μM).^183^ Similarly, **64**’s fragment was exploited to achieve bivalent ligand KN2 (**67**, Figure 20) bearing remarkable and selective inhibitory activities both toward CK2α-containing holoenzyme (*K*_i_ = 6.1 nM) and CK2α′-containing one (*K*_i_ = 4.0 nM). In this case, the same behavior and molecular plasticity of αD pocket within CK2α was also noted in its paralog CK2α′.^184^ With the aim to achieve a potent and pure allosteric CK2α inhibitor, several structural modifications were evaluated on both the biphenyl core and amino group of αD site ligand **65**. In this case, more extended compounds like CAM4712 (**68**, CK2α *K*_d_ = 3.0 μM, Figure 20) through the benzimidazole fragment forced Met163 flipping, thus blocking access to ATP site and CK2α kinase activity (CK2α IC_50_ = 7 μM).^185^ Noteworthy, allosteric inhibitor **68** demonstrated reduced selectivity in respect to bivalent ligand **66**. Another virtual screening campaign targeting αD site was recently reported identifying new uracil-based CK2 allosteric inhibitors.^186^ Furthermore, other several bivalent ligands targeting αD and ATP sites have been disclosed bearing promising (pre)­clinical efficacies, probing the potential of this approach in comparison to pure αD binders.^187^

**Figure 20:**
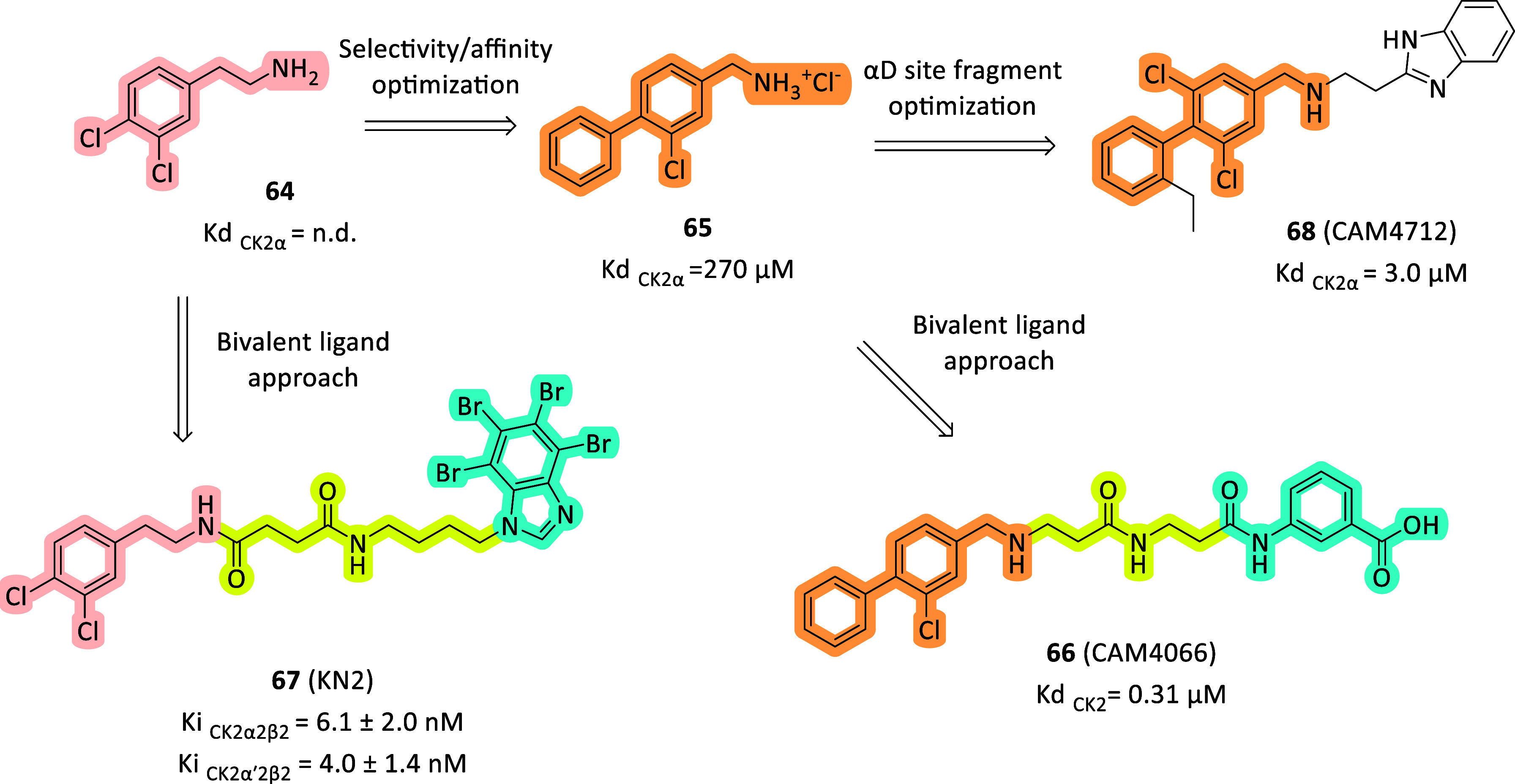
Chemical structures and biological activities of CK2 allosteric inhibitors targeting the allosteric pocket 2. In bivalent ligands it is highlighted in pink/orange the αD site binding moiety, the linker in yellow and in pale blue the ATP binding fragment.

Finally, allosteric pocket 3 was reported by Bestgen et al. in 2019, at the interface between αC helix and glycine rich loop. Once again, while looking for new ligands targeting the α/β interface of CK2, a new class of allosteric CK2 inhibitors was discovered.^188^ From a virtual screening campaign emerged compound **69** (CK2α IC_50_ = 27.7 μM, Figure 21), whose potency was later optimized achieving compound **70** (CK2α IC_50_ = 3.4 μM, Figure 21). Combination of STD/NMR, mutational mapping, competitive native mass spectrometry, and in silico docking localized the site of interaction of these 2-aminothiazoles at the interface of the two reported major lobes. In a further follow-up optimization, compound **71** (CK2α IC_50_ = 0.6 μM, Figure 21) was achieved as the most potent and selective allosteric CK2 inhibitors within this family.^189^ Surprisingly, later structural investigations from other groups on this class of compounds revealed an ATP-competitive mechanism and pointed at the orthosteric site as proper binding location, underlining the complexity of a proper and robust allostery characterization.^190^

**Figure 21:**
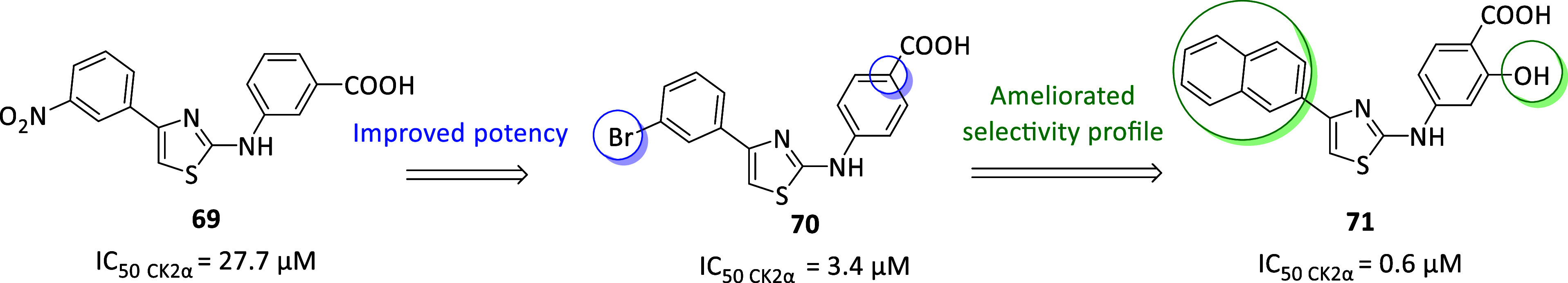
Chemical structures and biological activities of CK2 allosteric inhibitors targeting the allosteric pocket 3.

### Cell Division Cycle 7 Kinase: a Potential Hope for Future ALS Treatment

Cell division cycle 7 kinase (CDC7) is a Ser/Thr kinase that is involved in orchestrating DNA replication and cell cycle progression. It is mainly regulated by the interaction with an activation subunit called DBF4 that together with the kinase form the activated kinase complex,^191^ also called as DBF4 dependent kinase (DDK). CDC7-DBF4 is highly overexpressed in many cancer cell lines and primary tumors like ovarian, breast, liver, colon cancer, lung adenocarcinoma and melanoma,^192^ representing a prognostic marker and potential target in anticancer therapy with many inhibitors already in clinical trials.^193^ Furthermore, CDC7 resulted responsible for phosphorylation at Ser409 and Ser410 on TDP-43, a crucial step leading to its aggregation and neurotoxic accumulation resulting in one of the most consistent hallmarks of ALS and frontotemporal lobar dementia (FTLD).^194^ In this context, the relevant role of CDC7 in ALS and FTLD has been clarified with the development of brain permeable CDC7 inhibitors that have shown the ability to reduce the phosphorylation levels of TDP-43 in both human cell lines and transgenic ALS mice.^195^

To date, all of the reported CDC7 inhibitors carry an ATP-competitive mechanism, interacting in the catalytic site with the well-known issue of the lack of selectivity among the human kinome leading to undesired secondary effects. A first effort to identify non-ATP-competitive CDC7 inhibitors was conducted through screening of an FDA-approved drug library. Dequalinium chloride and Clofoctol (**72**, Figure 22), antimicrobial agents, were identified as CDC7 inhibitors targeting CDC7-DBF4 interaction with the resulting alteration of related pathways (e.g., MCM2 phosphorylation, DNA replication and delay cell cycle progression).^196^

**Figure 22:**
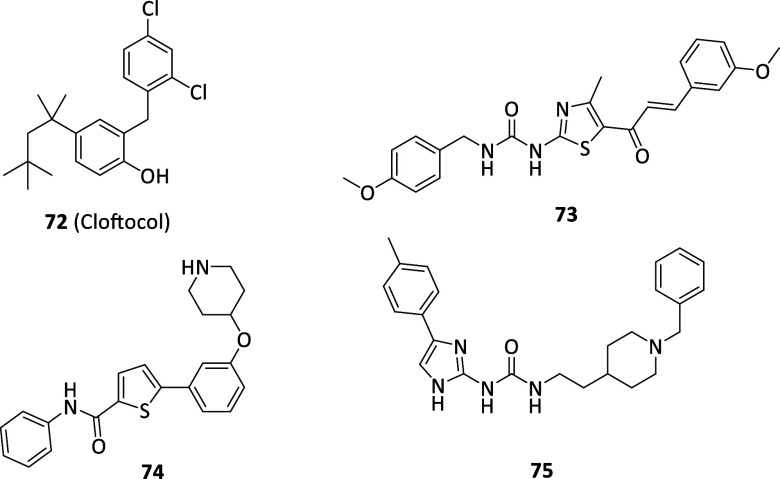
Chemical structures of CDC7 allosteric inhibitors.

With the aim to identify alternative CDC7 allosteric inhibitors, Rojas-Prats et al. first exploited different computational approaches to detect all possible druggable cavities on CDC7.^197^ Initially, a pocket finding campaign was performed using fpocket software:^198^ among the obtained 22 pockets, only 9 resulted druggable and present in all the examined CDC7 structures. Numbered from 1 to 9, pocket 1 is the catalytic site, pockets 2, 4, 6 and 9 include all the interaction sites between CDC7 and DBF4, while the other four conserved pockets (pockets 3, 5, 7, and 8) are located in different sites of the protein without participating in the direct interaction with the regulatory subunit. Particularly, structural analysis and site-directed mutagenesis experiments on CDC7-DBF4 interaction revealed Cys298, His309 and Val327 in motif C of DBF4 as essential for activity by binding and stabilizing the canonical αC helix of CDC7 comprehending the region covered by pockets 2 and 6. Therefore, further investigations were conducted only on these two cavities, which also turned out to be not well conserved among the 497 selected kinases. Previously identified allosteric inhibitor **72** was used as case study for a preliminary pocket validation: in silico studies proposed pocket 6 as interaction region occupying the site of α3 helix of DBF4 motif C binding to the protein through a hydrogen bond with Cys123’s backbone.

From a virtual screening of an in-house library on pockets 2 and 6 emerged eight putative allosteric binders which resulted in inactive in vitro, probably due to the hardness in breaking the CDC7-DBF4 interaction once formed in these conditions. Finally, in a cellular model only three compounds (**73**, **74**, **75**, Figure 22) were identified capable of impairing cellular pathways related to the inhibition of CDC7-DBF4 interaction (e.g., block DNA replication, delay cell cycle progression), deserving future extensive biological investigations.^197^

### Type 1 Insulin-like Growth Factor Receptor: Looking for Selectivity vs Insulin Receptor

The type 1 insulin-like growth factor receptor (IGF1R) is a tyrosine kinase receptor widely expressed in most vertebrate tissues and implicated in cell growth, development, and differentiation processes. IGF-1R is composed of 2 extracellular α-subunits, responsible of binding IGF, and two transmembrane β-subunits, hosting the intracellular Tyr kinase domain.^199^ IGF1R is activated by the extracellular binding of secreted growth factor peptides IGF-1, or IGF-2 and insulin with lower affinity, resulting in autophosphorylation of the intracellular kinase domain and phosphorylation of insulin receptor substrates 1/2 with the subsequent signaling cascade.^199^

IGF-1R is widely distributed throughout the CNS, particularly in neuronal precursor cells, with significant expression during cerebellar maturation and midbrain development, while IGF-1 supports neuronal development, cell survival, metabolism, and repair.^199^ Dysregulated IGF1 and IGF-1R levels were found under neurodegenerative conditions, although their role within pathogenesis is still unclear, because a lot of controversial outputs were reported. In AD IGF-1 improves nonamyloidogenic APP processing, prevents tau-phosphorylation and neuroinflammation, while elevated hippocampal and temporal IGF-1R levels were discovered in AD patients, higher serum IGF-1 is associated with increased PD risk, and IGF-1 levels correlate with cognitive dysfunction.^200^ Furthermore, genetically ablating IGF-1R signaling or IGF-1R inhibitor treatment foster neuroprotection and protect against AD progression, thus alleviating hallmarks such as Aβ deposition, neuroinflammation, neuronal and synaptic loss and behavioral dysfunction.^201^

Achieving selective IGF1R inhibitors for therapeutic purposes stands out particularly challenging because they share a high sequence identity of 84% in the tyrosine kinase domain with insulin receptor (IR). On the other hand, the inactivated and activated forms of IGF-1R and IR are structurally very different from each other, especially for the position of the activation loop and the αC helix. The only reported IGF-1R allosteric inhibitors originated from a HTS at Merck where compound **76** (Figure 23) was identified as first micromolar hit (IC_50_ = 10 μM) with an IC_50_ of 18 μM in cellular ELISA assays.^202^ Based on these premises, a SAR campaign was conducted achieving increased activity by shifting the amide junction from position 6 to 7 of the indole ring and introducing a cyano group in position 3 with electron-withdrawing properties (compounds **77** and **78**, Figure 23). Once verified the important selectivity versus insulin receptor, crystallography studies with compound **77** identified an adjacent binding pocket next to the DFG motif and the activation loop as its exact allosteric binding site. In this position the 5-cyano indole ring resulted sandwiched between Met1054 and Met1079 doing an H-bond between NH and the carbonyl group of Val1063 (Figure 23). Superimposing the cocrystal structure and apo form of IGF1R significant induced conformational changes were noticed in the activation loop, together with only moderate changes in the relative position of the GC loop and αC helix which can account for the inhibitory mechanism. Based on these pivotal results, several computational and structural analyses have been conducted to define at molecular level the structural framework which can set the base for further development of potent and selective IGF-1R inhibitors.^203^

**Figure 23:**
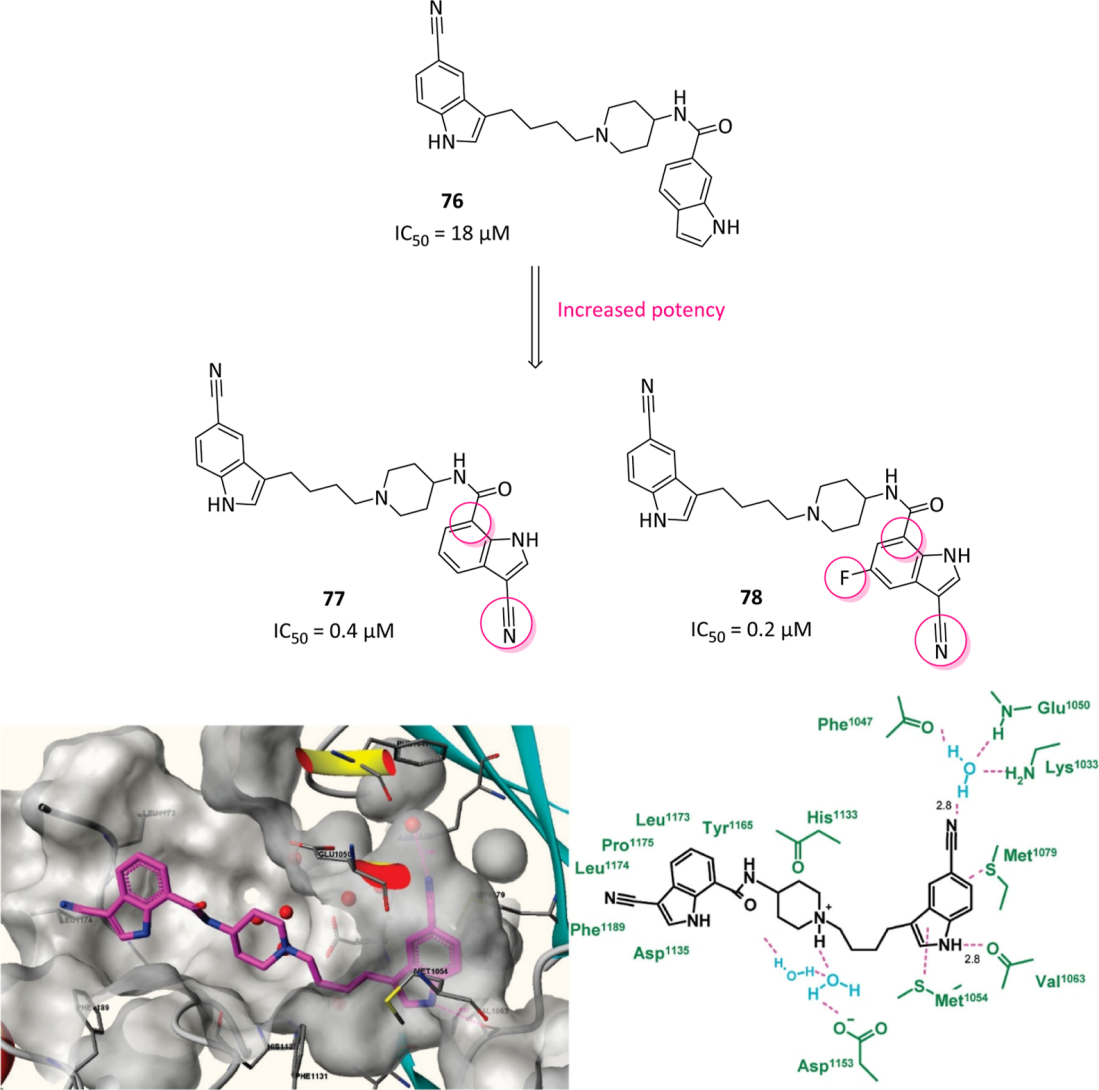
Chemical structures and biological activities of IGF1R allosteric inhibitors with the X-ray structure of **77** in the IGF1R allosteric binding site and the detailed interactions. Reprinted with permission from Heinrich, T.; Grädler, U.; Böttcher, H.; Blaukat, A.; Shutes, A. Allosteric IGF-1R Inhibitors. *ACS Med. Chem. Lett.* **2010**, 1 (5), 199–203. DOI: 10.1021/ml100044h.^202^ Copyright 2010 American Chemical Society.

### Tyrosine Kinase 2: the Benefit of Targeting the Pseudokinase Domain

Tyrosine kinase 2 (TYK2) is a nonreceptor tyrosine kinase, a member of the Janus kinase (JAK) family, which mediates intracellular signal transduction and cellular responses to growth factors and cytokines. Particularly, through the tuning of JAK/STAT pathway, it orchestrates the signaling of pro-inflammatory mediators such as TNF, IFN, IL-6, IL-10, IL-12 and IL-23.^204^ Differently to JAK1 and JAK2, TYK2-deficient mice are viable and considered resistant to collagen-induced arthritis (CIA) and EAE, while deactivating mutations in the Tyk2 gene could provide protection from multiple autoimmune disorders.^205^ For these reasons, TYK2 is considered an attractive target for autoimmune and inflammatory diseases and several nonselective JAKs inhibitors are already approved for the treatment of pathologies like myelofibrosis, rheumatoid arthritis and psoriasis.^206^

JAKs are composed of seven homology domains (JH) organized into four functional domains. A characteristic features in TYK2, like other JAK family members, is the presence of both a canonical catalytic kinase domain, called JAK homology 1 (JH1), and a pseudokinase domain catalytically inactive, called JAK homology 2 (JH2), proximal to each other.^207^ JH1 shares high degree of homology among JAKs, which then results in paucity of selective inhibitors related to several side effects in clinical studies (e.g., malignancy, thrombosis).^208^ JH2 structure bears higher specificity and demonstrates to exert regulatory functions on JH1 kinase domain, with JH2 inhibitors able to block it in the inactive conformation thereby preventing TYK2 activation.^209^ Recently, deucravacitinib (BMS-986165) represents the first selective TYK2 inhibitor (type VI) acting as JH2 binder approved by the FDA for psoriasis treatment.^210^

During the last years, TYK2 has emerged as pivotal regulator of neuroinflammatory processes and tau pathology, through phosphorylation at tau’s Tyr29, thus attracting interest for developing potential CNS therapies.^211^ Particularly, at the cellular level, TYK2 inhibition, by means of deucravacitinib or knockdown, mitigated total endogenous tau levels, while TYK2 overexpression increased overall tau burden. Albeit the complete lack of TYK2 led to primary immunodeficiency, partial suppression was beneficial and was proposed as strategy to reduce tau toxicity. In a tauopathy mouse model TYK2 knockdown reduced total and pathogenic tau species paired to attenuation of microgliosis and astrogliosis.^212^ Furthermore, in different animal models a centrally restricted TYK2 inhibitor completely rescued MS pathology and neuroinflammatory processes.^213^ Notably, after positively completed Phase I last year, an allosteric TYK2 inhibitor from Alumis Inc. (i.e., A-005) and one from Sudo Biosciences (WO2023227946A1) are planned to begin Phase II clinical trials in patients with MS in next months.^214^ Given that no structures or pharmacological characterizations of these candidates have been disclosed, clarifying the neuroprotective mechanism of action of allosteric TYK2 inhibitor at the cellular level, we decided to present TYK2 more as a prospective than validated target regarding allosteric modulation.

To date, the development of disclosed allosteric TYK2 inhibitors is mainly restricted to the hit-to-drug process leading to deucravacitinib carried out at Bristol-Myers Squibb (BMS) with some subsequent amendments.^215^ The history started with a phenotypic screening based on kinase inhibitors able to reduce IL-23/IFNα-derived inflammation, from which was derived compound **79** (Figure 24A) bearing submicromolar JH2 affinity and promising selectivity among a panel of around 380 kinases. Preliminary chemical refinements led to compound **80** (Figure 24A) showing increased functional potency, but paired to poor metabolic stability, modest pharmacokinetic properties and unwanted side activity (i.e., PDE4 inhibition).^216^ Later PK optimizations on the imidazopyridazine core achieved compound **81** (Figure 24A) which was orally active for the treatment of autoimmune and inflammatory diseased mice models. Limited space was found between C8 and the hinge region, confirmed by the loss of activity with substituents bulkier than methylamine. The replacement of an anilino moiety at C6 with 2-oxo-N1-substituted-1,2-dihydropyridin-3-ylamino group partially reduced the activity but notably increased metabolic stability and cell permeability. Finally, among tested aliphatic motifs, the (1*R*,2*S*)-2-fluorocyclopropyl amide in C3 greatly ameliorated the JH2 affinity while maintaining the desired PK profile.^217^ Recently, an unrelated drug discovery campaign conducted at Nimbus Therapeutics identified a similar pyrazolopyrimidine nucleus as the JH2 binder and TYK2 inhibitor. Multiple computational-based approaches were exploited to pinpoint methoxycyclobutyl amide moiety which boosted the binding affinity at JH2 domain in compound TAK-279 (**82**, Figure 24A). The crystal structure confirmed the perfect steric fit into the binding pocket defined by Val603 and Lys642 of TYK2 with the methoxycyclobutyl ring of **82**, while remained pivotal the two hydrogen bond networks: one between Val690 backbone and NH’s methylamino group in C8 and N1 of bicyclic core; the other occurred between C3′s carbonyl and amino group of Lys642 and carbonyl of Glu688 water-bridged (Figure 24B). Based on the important potency and selectivity, PK profile and determined biological properties, TAK-279 is currently in Phase II clinical trial (NCT06108544) for the treatment of psoriasis and psoriatic arthritis.^218^

**Figure 24:**
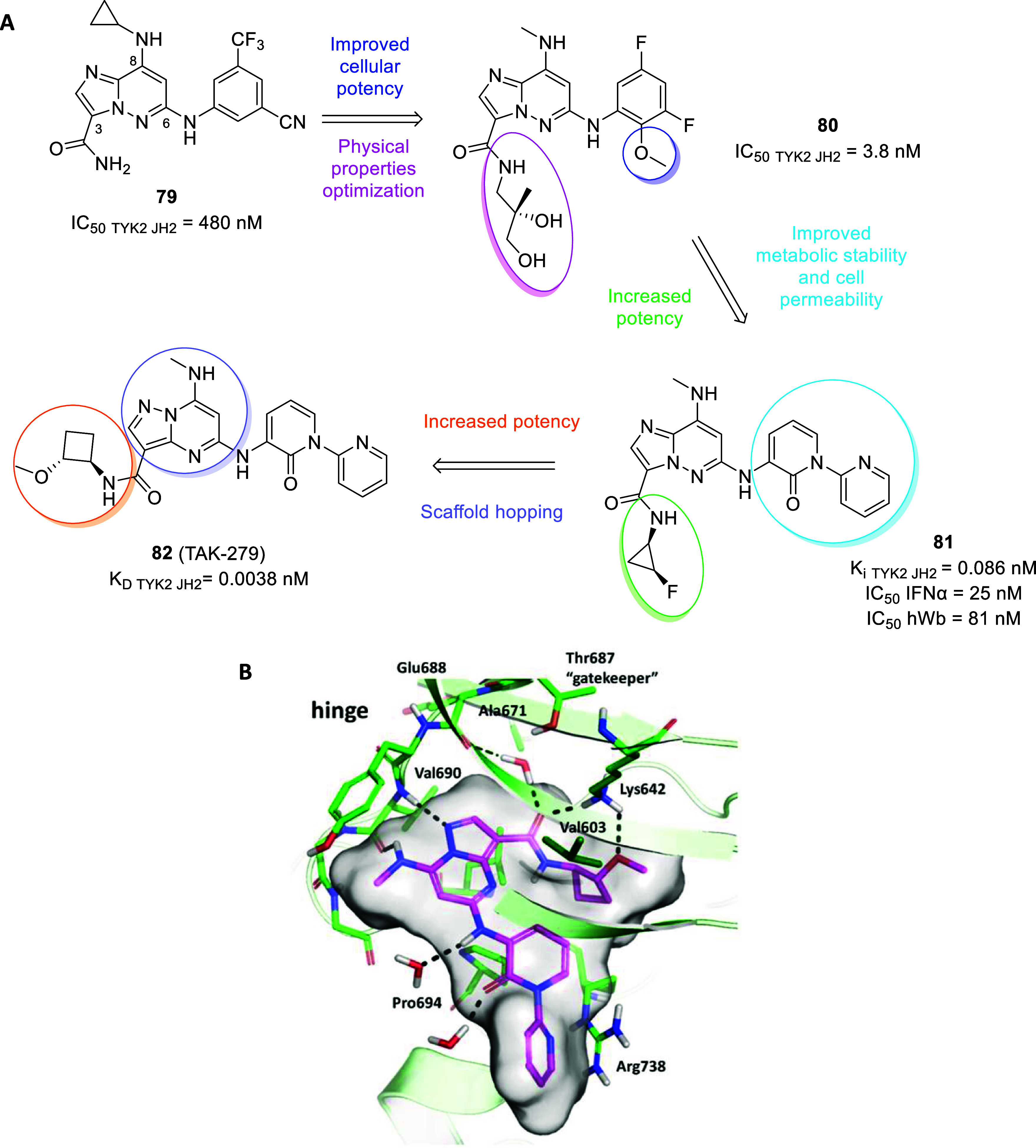
(A) Chemical structures and biological activities of TYK2 allosteric inhibitors (1). (B) Crystal structure of compound **82** bound to the JH2 domain of TYK2. Reprinted in part with permission from Leit, S.; Greenwood, J.; Carriero, S.; Mondal, S.; Abel, R.; Ashwell, M.; Blanchette, H.; Boyles, N. A.; Cartwright, M.; Collis, A.; et al. Discovery of a Potent and Selective Tyrosine Kinase 2 Inhibitor: TAK-279. *J. Med. Chem.* **2023**, 66 (15), 10473–10496. DOI: 10.1021/acs.jmedchem.3c00600.^218^ Copyright 2023 American Chemical Society.

In a parallel HTS, BMS characterized nicotinamide **83** (Figure 25A) as a more potent starting hit with respect to imidazopyridazine ones, albeit lacking selectivity. Insertion of a methyl in the C3 amide function significantly increased the selectivity among the kinome by binding to the atypical “alanine pocket” (Figure 25B), while deuterium incorporation ameliorated the metabolic stability due to reduced demethylation. Particularly, once anchored to the hinge region with a donor–acceptor–donor pattern, the methyl of amide in C3 was projected toward Ala671, which is present in this position only in other 9 kinases and is usually swapped with larger residues that would not normally tolerate a methyl group in this position. The pyridine-to-pyridazine conversion combined with the 2′ amide replacement with a methyl sulfonyl group improved potency and permeability, obtaining compound **84** (Figure 25A) which showed effective inhibition in an in vivo colitis mice model albeit exerting *h*ERG inhibition drawbacks.^219^ This last issue was settled through cyclopropylamide insertion in C6. Further modifications concerning the aniline moiety in 4, with a particular mention to the triazole group displacing an energetically unfavorable water molecule and engaging an additive H-bond with Arg738, increased the affinity for binding to JH2 domain led to compound BMS-986165 (**85**, Figure 25A), also known as deucravacitinib, which was approved in US and Australia in 2022 for psoriasis treatment.^210^ Further lead optimization focused on the side binding pocket revealed by the *N*-methyl triazole of **85**. From this emerged BMS-986202 (**86**, Figure 25A) showing a fluoropyrimidine instead of the triazole with the recurrence of the nicotinamide moiety. Overall, these modifications enabled the amelioration of aqueous solubility and cellular permeability. **86** successfully completed Phase I clinical trial (NCT02763969) and was proposed for psoriasis treatment.^220^

**Figure 25:**
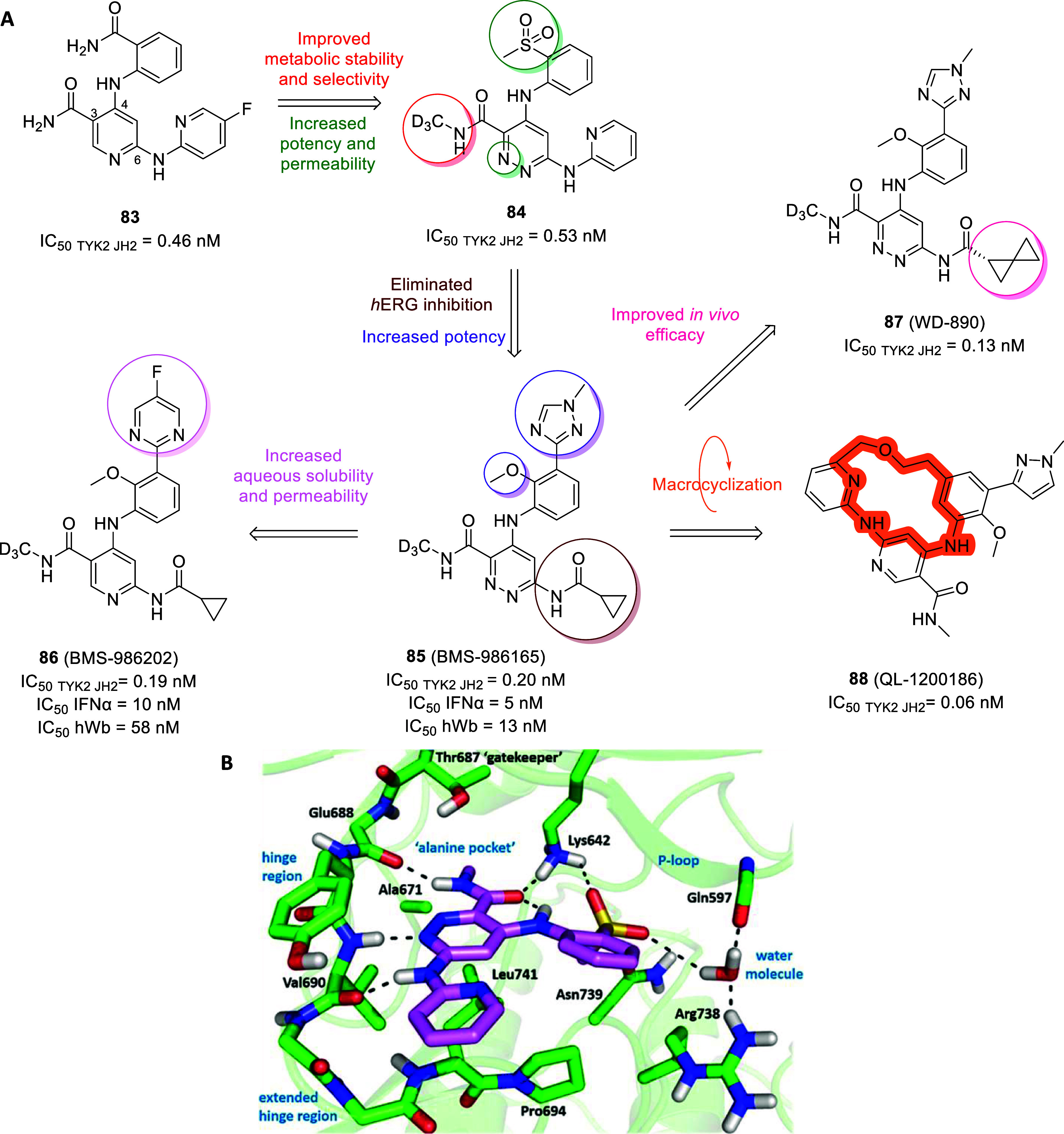
(A) Chemical structures and biological activities of TYK2 allosteric inhibitors (2). (B) Crystal structure of compound **84** complexed with TYK2 JH2. Reprinted with permission from Wrobleski, S. T.; Moslin, R.; Lin, S.; Zhang, Y.; Spergel, S.; Kempson, J.; Tokarski, J. S.; Strnad, J.; Zupa-Fernandez, A.; Cheng, L.; et al. Highly Selective Inhibition of Tyrosine Kinase 2 (TYK2) for the Treatment of Autoimmune Diseases: Discovery of Allosteric Inhibitor BMS-986165. *J. Med. Chem.* **2019**, 62 (20), 8973–8995. DOI: 10.1021/acs.jmedchem.9b00444.^210^ Copyright 2019 American Chemical Society.

Another two independent optimization campaigns were successively reported based on the pyridazine and nicotinamide core, respectively (Figure 25). Replacing the cyclopropylamide of BMS-986165 with a spiropentylamide resulted in compound WD-890 (**87**, Figure 25A) maintaining the same selectivity and potent inhibitory activity on TYK2.^221^ Favorable PK profile and therapeutic effects in several autoimmune disease animal models prompted **87** into a Phase I clinical trial (NCT06506591). On the other hand, macrocyclization strategy was applied onto previous reported nicotinamide derivatives to obtain QL-1200186 (**88**, Figure 25A) bearing greater selectivity profile and cellular potency in respect to **85** and **82**, respectively, while retaining similar therapeutic effects and PK properties.^222^

## Conclusions

In recent years the allosteric strategy turned out to be one of the most pursued alternative modalities to increase efficiency and selectivity in kinases’ modulation. In this Perspective, we reported the development routes of kinase allosteric modulators with already proved neuroprotective potential underlining successful approaches and pitfalls, with the aim to promote the progress of kinase allosteric modulators in this context and further enrich medicinal chemistry weaponry fighting neurodegeneration. To sum up the state of the art, for each kinase the most promising allosteric modulators bearing neuroprotective properties are reported in Table 1, highlighting the main pharmacological features in both preclinical and clinical studies, when known. In this regard, based on the herein described experimental workflows, several key points can be affirmed to highlight common strategies for achieving successful neuroprotective kinase allosteric modulators: (1) virtual or biochemical HTSs still remain the primary followed routes for allosteric hit identification; (2) generally, ranging from lipid or protein kinases to receptor-associated or nonreceptor kinases, the starting hit identified as allosteric modulator features great selectivity properties, especially when compared to the orthosteric analogues; (3) following hit-to-lead optimization procedures are mainly directed to ameliorate PK profile and BBB permeability due to the high degree of hydrophilicity commonly related to kinase ligands.

**Table 1:**
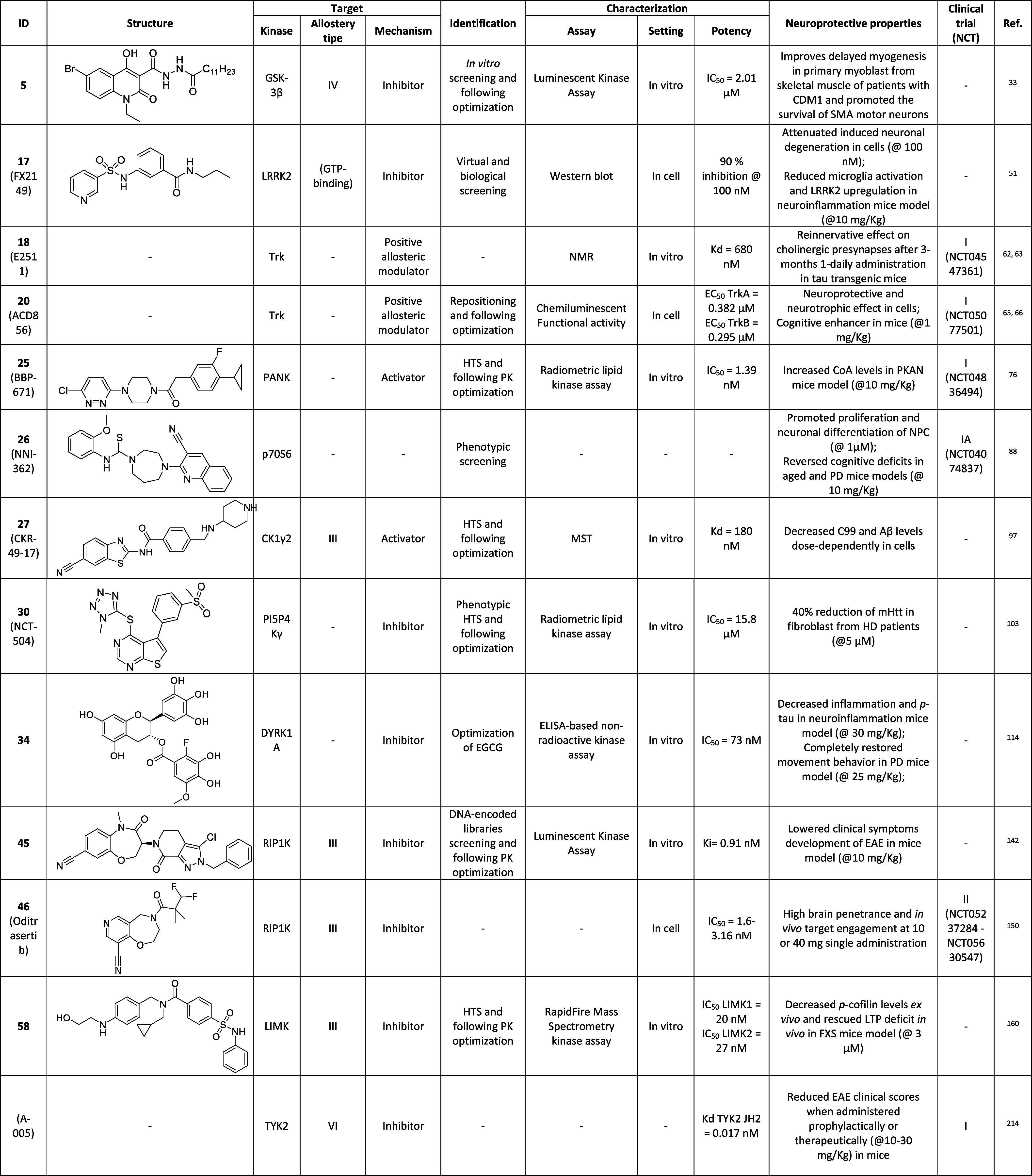
List of Most Promising Allosteric Kinase Modulators Bearing Neuroprotective Properties With Their Main Features

As demonstrated by the first three examples of non-CNS allosteric drug discovery campaigns, allosteric modulation may be the answer to the main issues for kinase inhibitors’ clinical translation. In many cases, allosteric inhibitors allowed us to definitely overcome main drawbacks of orthosteric ones thanks to the reached selectivity. Functional selectivity solved toxic concerns derived from common prolonged orthosteric inhibition (e.g., GSK-3β, LRRK2), resulted in CNS-selective action (e.g., p70S6) or provided a more effective and specific neuroprotective efficacy (e.g., Trk, PANK). The achieved kinome, or even isoform, selectivity was relevant to univocally define roles of specific kinases (e.g., PI5P4Kγ, RIPK1), while in other case the respective allosteric inhibitors might be the key to the wanted subtype-selectivity, albeit still to be confirmed (e.g., CK1γ2, DYRK1A). Notably, the allosteric LIMK inhibitor **57** highlighted a rare case of an absolute selectivity profile.

## Major Issues and Potential Fixes

Case studies faced herein have pointed out two intrinsic major challenges for this approach: robust allosteric pocket identification and validation procedures paired with the usual CNS bioavailability issues. To overcome this latter, several medicinal chemistry strategies (e.g., prodrug, tuning lipophilicity, carrier linking) can be applied and turned out to be efficient even for CNS-directed kinases ligand development.^223^ As a confirmation of facts, from a literature review on brain penetrant kinase inhibitors, properly “CNS designed” kinase inhibitors resulted featuring median physicochemical properties in a similar extent to CNS drugs, although this was not sufficient for any clinical translation.^5^ However, allosteric pockets are generally more hydrophobic than the orthosteric sites and, as result, allosteric modulators tend to be more rigid and lipophilic, defining a different starting point toward CNS kinase allosteric ligands optimization.^224^ Alternatively, tailored strategies for a brain-selective kinase modulation can rely on the specificity of action of the intended ligand/target to restrict its activity at the central level. In this regard, an elegant strategy was developed for a rapamycin derivative acting as a CNS-directed mTOR inhibitor. Given that it required the protein FKBP12 for its intracellular activity, the coadministration of mTOR inhibitor with a potent and peripherally restricted FKBP12 ligand allowed brain-specific inhibition of mTOR with showed efficacy in glioblastoma models, while mitigating undesired systemic effects.^225^ Evidently, however this approach is confined to those target/ligand of which there is a deep knowledge regarding the working mechanisms.^226^

Many shortcomings are related to allosteric pocket identification/characterization procedures, as exemplified by the above-reported cases of virtual screening campaigns focused on specific allosteric pocket, which then provided allosteric modulators but acting on different/vicinal site. Noteworthy, among the years several computational tools were developed aiming to efficiently detect allosteric sites with a resulting boost in allosteric modulators identification.^227^ Particularly, allosteric site detection, communication and related modulators’ screening/design software provided robust aid in allosteric ligand development in respect to experimental-only approaches.^228^ In this context, artificial intelligence and machine learning tools may further foster allosteric drug development in the near future.^229^ However, experimental binding and functional activity assays together with biophysical methods for binding site identification are essential to properly validate kinase allosteric modulators.^11^ In this respect, kinetic experiments can be challenging because of competitive exceptions or slow binding phenomena depending on the specific allosteric mechanism, as already noted above. Therefore, the iterative workflow between bioinformatic and biophysical/biochemical methods has proven to be one of the most effective strategies for this purpose.^230^ Normally, one of the main straightforward tactics turned out to be an initial allosteric site prediction computationally driven following by the experimental validation based on point mutation analysis, nuclear magnetic resonance or kinetic assays.^231^ To note, the same accurate proceeding should be exploited for allosteric binders characterization to avoid false readouts.^188^

## Future Perspectives

Despite these important premises, the hunt for allosteric drugs is still slow. Albeit many strategies proved their efficiency in overcoming CNS-targeting issues, the identification of allosteric modulators remains the main hurdle. A plethora of computational techniques have been developed for predicting allosteric mechanisms, but the consequent experimental validation protocols present several flaws. Nowadays, setting up experimental assays and developing valid chemical probes for measuring allosteric modulation and subsequent signaling are of utmost importance.

The majority of allosteric compounds reported herein were initially identified by chance or via high-throughput screening. Therefore, implementing alternative screening strategy (e.g., NMR or X-ray crystallography platform, fragment-based approaches) is another top priority in allosteric drug discovery campaigns.

Moreover, the history of certain approved kinase allosteric inhibitors demonstrates that the path to success is easier when validated tool compounds are available.^11^ Leveraging these advantages becomes of paramount importance in drug development pathways with already the highest attrition rate toward clinical application (i.e., CNS drug discovery). Therefore, in addition to canonical allosteric kinase modulators, targeting kinase allosteric sites with alternative chemical modalities can provide valuable avenues for future CNS pharmacological tools. Particularly, efficient and prolonged modulation by means of allosteric covalent ligands allows a deep mechanistic understanding of the allosteric pocket’s involvement regarding the kinase functional activity, while allosteric-based degraders can define the kinase role in neurodegenerative processes with high sensitivity at the cellular level. To note, merging the pharmacological merits of allosteric modulation with both modalities should dramatically increase the specificity of action which represents one of the essential requirements for validated chemical probes.^232^ Covalent allosteric kinase inhibitors are already in advanced preclinical studies (e.g., borussertib as AKT inhibitor for cancer treatment), thus probing their potential,^233^ while degraders based on allosteric kinase ligands are gaining increasing interest.^234^

In this article we also highlight some examples of understudied kinases for neuroprotective purposes so far, characterized by already detected allosteric pockets and, most importantly, demonstrated involvement in neurotoxic pathways. The struggles of neuroscience are mainly due to poor knowledge regarding the intertwined mechanisms underlying neurodegeneration, and the investigation of alternative targets with benefits of allosteric modulation may lead to new promising perspectives. Furthermore, besides canonical kinases, among the neural kinome there is still a subset of underexplored kinases linked to neurodegenerative diseases, whose study may open to potential new targets for CNS drugs.^235^ Based on all of these aspects, the allosteric path can be much trickier but has to be faced because it could dramatically change kinase drug discovery campaigns for CNS disorders.

## Abbreviations

AcCoA: Acetyl-Coenzyme A
ACh: acetylcholine
AD: Alzheimer’s disease
ADME: absorption distribution metabolism excretion
ALS: amyotrophic lateral sclerosis
APP: Amyloid Precursor Protein
Aβ: β-: Amyloid peptide
ATP: Adenosine triphosphate
BBB: blood brain barrier
BDNF: brain-derived neurotrophic factor
BMS: Bristol-Myers Squibb
BTO: benzothiazinone
BTZ: benzothiazepinone
CDC7: Cell division cycle 7 kinase
CDK: Cyclin-Dependent Kinase
CDM1: congenital myotonic dystrophy type 1
CK: casein kinase
CIA: collagen-induced arthritis
CML: chronic myelogenous leukemia
CNS: central nervous system
CSF: Cerebrospinal Fluid
DD: death domain
DDK: DBF4 dependent kinase
DBCA: N: *N*-dibenzylcinnamamide
DYRKs: Dual-specificity tyrosine-phosphorylation regulated kinases
DS: Down Syndrome
EAE: experimental autoimmune encephalomyelitis
EGCG: epigallocatechin gallate
EGFR: epidermal growth factor receptor
ERK: Extracellular signal-Regulated Kinase
FADD: Fas-Associated protein with Death Domain
FTLD: frontotemporal lobar dementia
FXS: Fragile X Syndrome
GEF-CK: Golgi-enriched fraction CK
GSK-3β: Glycogen synthase kinase-3β
GTP: Guanosine Triphosphate
HD: Huntington’s disease
Htt: huntingtin
HTS: high throughput screening
IFN: interferon
IGF1R: type 1 insulin-like growth factor receptor
IL: interleukin
JAK: Janus kinase
JH: JAK homology
LGMDR1: limb girdle muscular dystrophy R1 calpain 3-related
LRRK2: Leucin-rich repeat kinase 2
LPS: lipopolysaccharide
MAPK: Mitogen-Activated Protein Kinase
MEK: mitogen-activated protein kinase kinase
MLKL: mixed lineage kinase domain-like protein
MRCK: Myotonic dystrophy kinase–related Cdc42-binding kinase
MS: Multiple Sclerosis
NFT: neurofibrillary tangles
NGF: nerve grow factor
NSCLC: nonsmall cell lung cancer
NTs: neurotrophins
PAK: p21-activated kinase
PANK: Pantothenate kinase
PKAN: pantothenate kinase-associated neurodegeneration
PDB: protein data bank
PD: Parkinson’s disease
PI3K: Phosphoinositide 3-kinase
PI4: 5P2: phosphatidylinositol 4,5-bisphosphate
PI5P: phosphatidylinositol 5-monophosphate
PI5P4Ks: phosphatidylinositol 5-phosphate 4-kinases
PK: pharmacokinetic
POM: polyoxometalates
PPI: protein–protein interaction
PS1: presenilin 1
p70S6: p70 ribosomal S6 kinase
RHIM: RIP Homotypic Interaction Motif
RIPK: Receptor-interacting protein kinases
ROCK: Rho-associated coiled-coil containing kinase
ROS: Reactive Oxygen Species
SMA: spinal muscular atrophy
TKL: tyrosine kinase-like family
TDP-43: transactive response DNA binding protein of 43 kDa
TNF-α: tumor necrosis factor α
Trk: Tropomyosin receptor kinases
TYK2: Tyrosine kinase 2
WT: wild type
